# Sporothioethers: deactivated alkyl citrates from the fungus *Hypomontagnella monticulosa*†

**DOI:** 10.1039/d3ra06542a

**Published:** 2023-10-11

**Authors:** Henrike Heinemann, Kevin Becker, Hedda Schrey, Haoxuan Zeng, Marc Stadler, Russell J. Cox

**Affiliations:** a OCI, BMWZ, Leibniz University of Hannover Schneiderberg 38 30167 Hannover Germany russell.cox@oci.uni-hannover.de; b Department Microbial Drugs, Helmholtz Centre for Infection Research (HZI) Inhoffenstraße 7 38124 Braunschweig Germany; c Institute of Microbiology, Technische Universität Braunschweig Spielmannstraße 7 38106 Braunschweig Germany

## Abstract

Submerged cultivation of *Hypomontagnella monticulosa* MUCL 54604 resulted in formation of a stereoisomeric mixture of new sulfur-containing sporothriolide derivatives named sporothioethers A and B. The presence of the 2-hydroxy-3-mercaptopropanoic acid moiety attenuates the antimicrobial activity in comparison to the precursor sporothriolide suggesting a detoxification mechanism. However, moderate effects on biofilms of *Candida albicans* and *Staphylococcus aureus* were observed for sporothriolide and sporothioethers A and B at concentrations below their MICs.

## Abstract 2

Two new thioether specialised metabolites isolated from the fungus *Hypomontagnella monticulosa* show attenuated antifungal properties, but retain biofilm inhibition activity.

## Graphical abstract

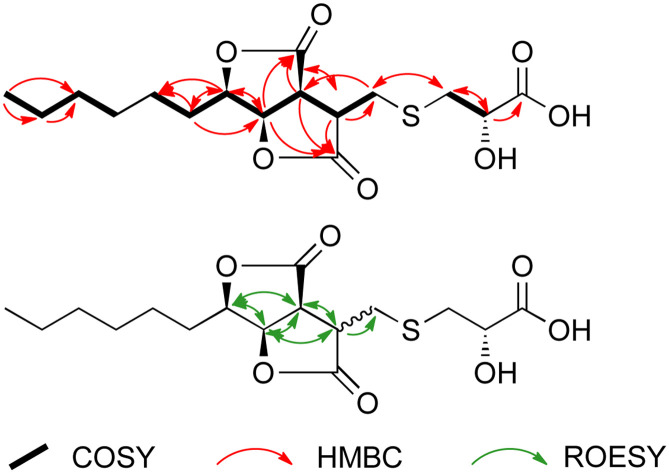

Alkyl citrates are a structurally broad class of natural products requiring an alkyl citrate synthase (ACS) for their biosynthesis.^1^ Examples of fungal alkyl citrates (Scheme 1A) include byssochlamic acid 1,^2,3^ the potent squalene synthase inhibitor squalestatin S1 2,^4,5^ and piliformic acid 3.^6^ We recently fully characterised the biosynthetic pathway to sporothriolide 8 that is an alkyl citrate isolated from *Hypomontagnella* spp.^7–9^ The *spo* biosynthetic gene cluster (BGC) is responsible for production of 8 (Scheme 1B).^10,11^ Decanoyl-CoA, produced by a dedicated fatty acid synthase (FAS), is used by the ACS SpoE to form the alkyl citrate 4. Subsequent enzymatic dehydration to 5, decarboxylation to 6, hydroxylation to 7 and lactonization then forms the furofurandione sporothriolide 8 (Scheme 1B). Spontaneous Diels–Alder (DA) reaction with the polyene trienylfuranol A 9, then results in formation of the sporochartines 10–13 (Scheme 1B). Sporothriolide 8 itself possesses useful antifungal activity.^9^ For example it has been shown to protect pepper seedlings from the plant pathogen *Botrytis cinerea*.^12^ Sporochartines A-D 10–13, in turn, are potent cytotoxins active against human cancer cell lines.^11^

**Scheme 1:**
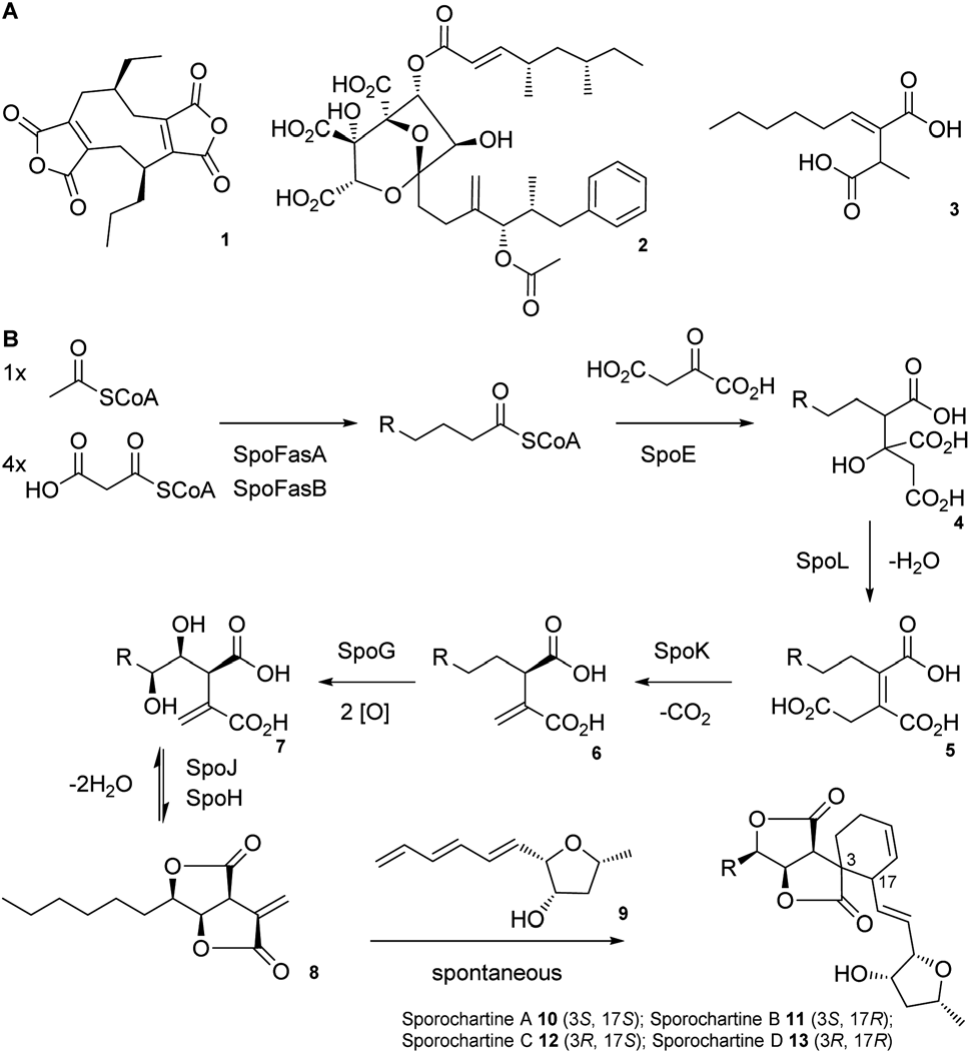
A) Structures of alkyl citrate metabolites from fungi; (B) biosynthesis of sporothriolide 8 and sporochartines 10–13. R = C_6_H_14_.

Here, we report the isolation, synthesis, structure elucidation, and biological testing of two new sulphur-containing sporothriolide derivatives, termed sporothioether A 14 and B 15 (Fig. 1) from *H. monticulosa* MUCL 54604.^10^

**Fig. 1:**
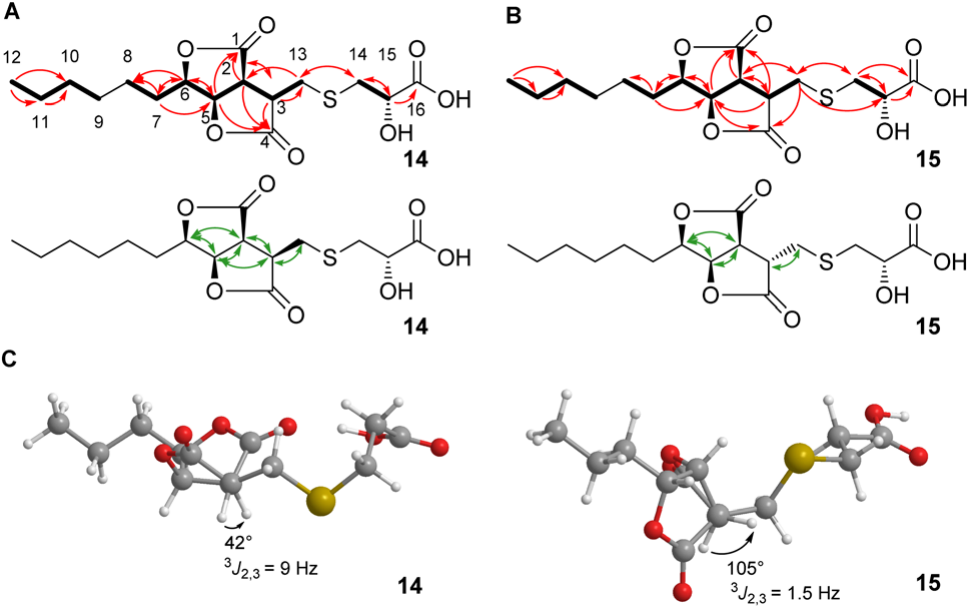
Key NMR information. (A) COSY correlations (bold lines), HMBC correlations (red arrows) and ROESY correlations (green arrows) of sporothioether 14; (B) COSY correlations (bold lines), HMBC correlations (red arrows) and ROESY correlations (green arrows) of sporothioether 15; (C) energy-minimized model structures of sporothioethers 14 and 15 used for a *J*-based assignment of relative stereochemistry.

In previous work, sporothriolide 8 was isolated from extracts of *H. monticulosa* MUCL 54604 after cultivation in PDB medium.^10^ However, when *H. monticulosa* was cultivated in DPY medium, sporothriolide 8 was not detected. DPY was therefore previously used as a non-producing condition during transcriptomic analysis of the *spo* BGC.^10^ However, analysis of the transcriptomic data did not reveal significant down-regulation of the *spo* genes in DPY media, and LCMS analysis of the culture extract revealed the presence of a new metabolite under these conditions. Purification of the new metabolite using preparative reversed-phase chromatography (ESI, Fig. S1†) resulted in isolation of an inseparable 3 : 2 mixture of isomers 14 and 15 (as indicated by ^1^H NMR integrals, ESI, Fig. S5†). A measured *m*/*z* of 361.1316 [M + H]^+^ (ESI, Fig. S2†) suggested a molecular formula of C_16_H_24_O_7_S for each compound. Methylation with TMS-diazomethane led to formation of a 3 : 2 mixture of the corresponding methyl esters 16 and 17 (*m*/*z* 373 [M − H]^−^), indicating the presence of a carboxyl group in 14 and 15 (ESI, Fig. S3†).

In the ^1^H-NMR and HSQC spectra of the 14 and 15 mixture (Table S1†) peaks for a methyl group (*δ*_H_ 0.93 ppm), seven methylenes (*δ*_H_ 1.34, 1.35, 1.41, 1.50, 1.81, 3.00/(3.08; 2.88), 3.06/(3.24; 2.96)), four methines (*δ*_H_ 3.66/3.17, 3.84/3.79, 4.63/4.71, 5.14/5.28) and a hydroxymethine (*δ*_H,_ 4.36/4.34) were observed. All peaks were doubled in a 3 : 2 ratio. The ^13^C data revealed two sets of 16 carbons. Three of the 16 carbons of each set are carbonyls (*δ*_C_ 176.4/176.3, 174.6/177.5, 176.1/177.0), seven are methylene carbons (*δ*_C_ 23.5, 26.3, 29.8, 29.8/34.3, 30.0, 32.7, 38.4/37.1), one is a methyl carbon (*δ*_C_ 14.2) and five are methines (*δ*_C_ 44.8/45.6, 45.0/47.7, 72.3, 80.4/81.3, 82.5/83.6).

2D NMR spectra (ESI, Fig. S7–S10†) showed the core structures of 14 and 15 to be the sporothriolide scaffold (Fig. 1A and B). In addition to this core structure, both new compounds harbour a 2-hydroxy-3-mercaptopropanoic acid moiety attached to C-13 of the core *via* a thioether bond. This linkage was supported by the HMBC correlation between CH_2_-14 and C-13 (Fig. 1A and B). The presence of the thioether was additionally confirmed by the distinctive ^1^H/^13^C chemical shifts of C-13 (14, *δ*_H_ 3.06 and *δ*_C_ 29.8; 15, *δ*_H_ 3.24/2.96 and *δ*_C_ 34.3) and C-14 (14, *δ*_H_ 3.0 and *δ*_C_ 38.4; 15, *δ*_H_ 3.08/2.98 and *δ*_C_ 37.1). However, the stereochemistry at position C-3 differs in sporothioether A 14 and B 15. ROESY correlations (Fig. 1, A, B) showed that H-3, H-2, H-5 and H-6 are all *syn* in 14, but in 15 H-3 is *anti* to H-2, H-5 and H-6, as no ROESY correlation is observed. An energy-minimised model structure of the *anti* diastereomer 15 suggested a dihedral angle of 105° for H-3/H-2 consistent with the observed ^3^*J*_2,3_ value of 1.5 Hz (Fig. 1C). In contrast, an energy-minimised model of diastereomer 14 has a dihedral angle of 42° consistent with the observed ^3^*J*_2,3_ value of 9 Hz (Fig. 1C). Assuming the same absolute stereochemistry at C-2, C-5 and C-6 as in the parent compound sporothriolide 8, we conclude that in *syn* diastereomer 14, C-3 is *R*-configured, and in *anti* diastereomer 15, C-3 is *S*-configured.

In order to determine the configuration of the stereocenter at C-15 we initially attempted to form Mosher's esters of the 15-OH.^13^ However, this approach led to inconclusive results due to the complexity of the spectra of the resulting mixed diastereomers. In an alternative strategy, 15*S* and 15*R* sporothioether derivates were chemically synthesized for comparison by NMR spectroscopy (Scheme 2A).

**Scheme 2:**
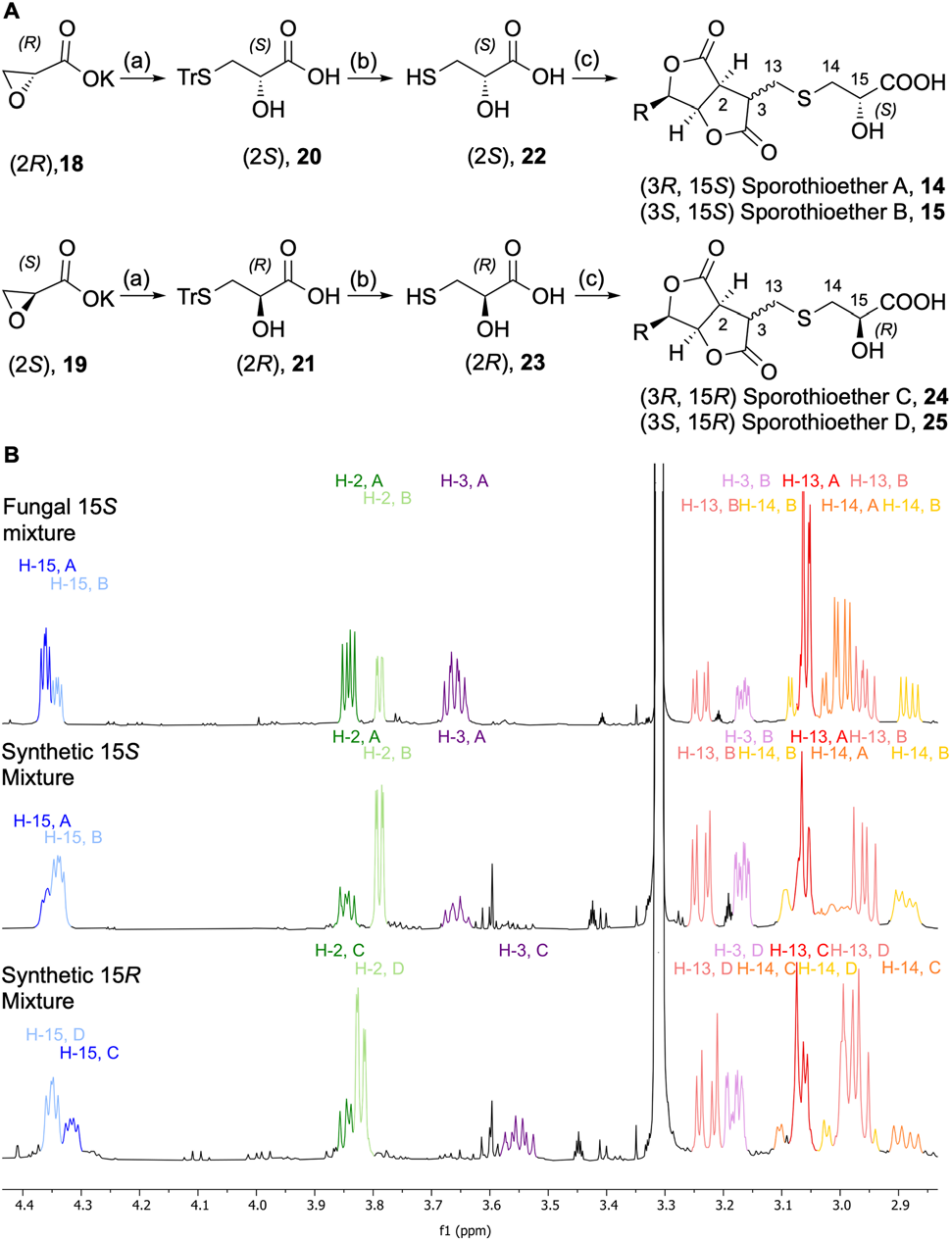
Synthesis and analysis of sporothioether derivates. (A) Synthetic route towards sporothioethers 14–15 and 24–25: (a) TrSH (1.3 eq.), NaH (1.3 eq.), THF, 0 °C-RT, overnight, (b) Et_3_SiH (3 eq.), TFA, DCM, 30 min, (c) Sporothriolide 8 (0.8 eq.), Et_3_N (5 eq.), CH_2_Cl_2_, RT, 2 h; Tr = triphenylmethyl; (B) comparison of ^1^H NMR data of fungal sporothioethers A 14 and B 15, semisynthetic sporothioether A 14 and B 15 and semisynthetic sporothioether C 24 and D 25.

Commercially available oxirane carboxylate enantiomers 18 and 19 were subjected to regioselective ring opening over two steps by treatment with triphenylmethanethiol to give intermediates 20 and 21, followed by acidic deprotection to give 22 and 23. Sporothriolide 8 was purified from *H. monticulosa* and reacted under basic conditions with either 22 or 23. The resulting sporothioethers were purified by reversed-phase chromatography as inseparable mixtures of diastereomers in each case.

Analysis of the ^1^H NMR data (Scheme 2B) of the synthetic products revealed that both contain a mixture of sporothioether stereoisomers, as indicated by two sets of signals with an integral ratio of 3 : 2. The major compound in both mixtures is 3*S* and the minor component is 3*R*. This was indicated by the ^3^*J*_2,3_ value of 1.6 Hz for the 3*S* configured sporothioethers. For the 3*R* configured stereoisomers ^3^*J*_2,3_ values of ∼9 Hz were observed, the same as for the natural 3*R* sporothioether 14. ^1^H NMR chemical shifts of the synthetic (15*S*, 3*RS*)-sporothioether mixture matched the signals of the fungal sporothioether mixture 14 and 15, whereas the signals of the synthetic (15*R*, 3*RS*)-sporothioether mixture 24 and 25 differed (Scheme 2B). Therefore, sporothioether A 14, the major compound in the natural mixture, is identified as (3*R*, 15*S*) and the minor compound sporothioether B 15 is identified as (3*S*, 15*S*). The non-natural synthetic diastereomers were now named sporothioether C 24 (3*R*, 15*R*) and sporothioether D 25 (3*S*, 15*R*).

It seems likely that the sporothioethers are biosynthesised by addition of 2*S*-2-hydroxy-3-mercaptopropanoic acid 22 to sporothriolide 8 itself *in vivo*. Formation of an epimeric mixture at C-3 may indicate that this is not an enzymatic process. To test this hypothesis sporothriolide 8 was incubated with 3-mercaptopropionic acid 26 under physiological conditions in phosphate buffer at pH 7.5. LCMS analysis revealed the formation of a new compound with an *m*/*z* of 343 [M − H]^−^, which corresponds to the mass of the expected product 30 and 31 (ESI, scheme S1†).

The inseparable mixtures of 14 and 15, and 24 and 25, were assayed for biological activity (see ESI†). In cytotoxicity assays no activity was detected for the sporothioethers against KB-3-1 and L929 cell lines in the tested range (37–0.63 μg mL^−1^). Similarly, in previous studies no cytotoxic activity was detected for sporothriolide 8 itself.^9^ Both, the sporothioether mixture 14 and 15 and sporothriolide 8, were tested in biofilm inhibition and dispersion assays against *Stapylococcus aureus* and *Candida albicans* (Table 1). Sporothriolide 8 displayed significant activity against the formation of *S. aureus* biofilms at subtoxic concentrations (MIC (*S. aureus*) > 66.6 μg mL^−1^), meanwhile only weak inhibitory effects on preformed biofilm of *S. aureus* were observed.

**Table 1:** Inhibition of biofilm formation of *Staphylococcus aureus* and *Candida albicans* and dispersion of preformed biofilms of *S. aureus* by sporothioether mixture (14 and 15) and sporothriolide 8 at various concentrations; references [%]

Test organism		14 + 15 mixture	8
Biofilm inhibition [% ± SD]	*S. aureus* (DSM 1104)^a^	77 ± 9 (250 μg mL^−1^)	75 ± 8 (250 μg mL^−1^)
		24 ± 9 (62.5 μg mL^−1^)	55 ± 9 (3.9 μg mL^−1^)
			37 ± 10 (2 μg mL^−1^)
	*C. albicans* (DSM 11225)^b^	58 ± 8 (250 μg mL^−1^)	—
		40 ± 10 (31.3 μg mL^−1^)	
Biofilm dispersion [% ± SD]	*S. aureus* (DSM 1104)^c^	77 ± 9 (250 μg mL^−1^)	53 ± 6 (250 μg mL^−1^)
		24 ± 9 (62.5 μg mL^−1^)	36 ± 5 (62.5 μg mL^−1^)

a Microporenic acid A, 74 ± 12 (250 μg mL^−1^), 75 ± 6 (7.8 μg mL^−1^), 42 ± 7 (3.9 μg mL^−1^).

b Farnesol: 75 ± 9 (250 μg mL^−1^), 51 ± 7 (31.3 μg mL^−1^), 38 ± 10.

c Microporenic acid A: 64 ± 7 (250 μg mL^−1^), 41 ± 15 (15.6 μg mL^−1^); SD: standard deviation; —no inhibition.

In addition, the sporothioether mixture 14 and 15 displayed moderate inhibitory activity against biofilm formation of *C. albicans* (Table 1). In antimicrobial assays the mixtures of 14 and 15 and 24 and 25 were tested against selected bacteria and fungi in the same assays that were used previously to assess the bioactivity of sporothriolide 8.^9^ The minimum inhibitory concentrations (MIC, Table 2) showed that sporothriolide 8 possesses moderate antimicrobial activity against the tested microorganisms as reported previously,^9^ but sporothioethers 14 and 15 were inactive against most organisms and had significantly attenuated effects *vs. Mucor hiemalis* and *Schizosaccharomyces pombe*. Sporothioethers 24 and 25 were inactive against all tested microorganisms.

**Table 2:** Minimal inhibitory concentrations (MIC) of sporothioether mixtures (14 and 15; 24 and 25) dissolved in MeOH, sporothriolide 8 and control drugs

Test organism	MIC [μg ml^−1^]			
	14 and 15 mix	24 and 25 mix	8 (ref. 9)	Ref.
*Schizosaccharomyces pombe* (DSM 70572)	66.6	—	8.3	4.2^a^
*Pichia anomala* (DSM 6766)	—	—	33.3	8.3^a^
*Mucor hiemalis* (DSM 2656)	66.6	—	4.2	4.2^a^
*Candida albicans* (DSM 1665)	—	—	16.6	8.3^a^
*Rhodotorula glutinis* (DSM 10134)	—	—	16.6	2.1^a^
*Acinetobacter baumannii* (DSM 30008)	—	—	nt	0.26^b^
*Escherichia coli* (DSM 1116)	—	—	—	1.7^c^
*Bacillus subtilis* (DSM 10)	—	—	—	8.3^c^
*Mycobacterium smegmatis* (ATCC 700084)	—	—	nt	1.7^d^
*Staphylococcus aureus* (DSM 346)	—	—	—	1.7^c^
*Pseudomonas aeruginosa* (PA 14)	—	—	—	0.42^e^
*Chromobacterium violaceum* (DSM 30191)	—	—	nt	0.42^c^

a Nystatin.

b Ciprobay.

c Oxytetracyclin hydrochloride.

d Kanamycin.

e Gentamycin; – no inhibition; nt, not tested. The cell density was adjusted to 8 × 10^6^ cells per ml.

The sporothioethers 14 and 15 appear to arise by spontaneous addition of 2*S*-2-hydroxy-3-mercaptopropionate to sporothriolide. This is supported by observation of facile addition of 3-mercaptopropionate to 8 in the absence of biological catalysts. This is also consistent with the *S*-configuration of the 2-hydroxy-3-mercaptopropanoic acid moiety in other natural compounds, such as berkeleylactone A 28,^14^ sumularin C 30,^15^ and thiopleurotin 32 (Fig. 2).^16^

**Fig. 2:**
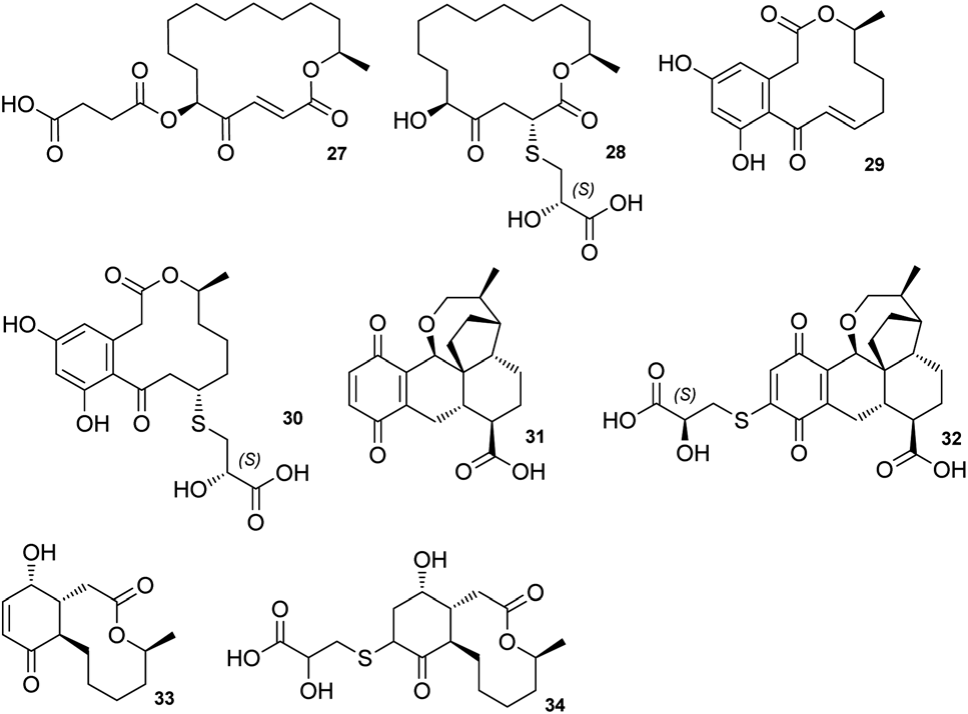
Structures of metabolites containing the 2-hydroxy-3-mercaptopropanoic acid moiety and their congeners.

For the latter compound, cysteine was identified as the origin for the sidechain 22 and this is also likely to be the case for the sporothioethers.^16,17^ In our hands the 3-epimeric forms of the sporothioethers were inseparable. However, it is clear that material isolated from biological and synthetic sources contains differing proportions of the epimers (Scheme 2B). This may be explained by preferential extraction, or preferential degradation, of one epimer from the biological source.

Some of the 2-hydroxy-3-mercaptopropanoic acid moiety-bearing natural compounds have potent bioactivities, whereas others are significantly less toxic in comparison to their originating compounds. For example, sumalarin C 30 is a potent cytotoxin *vs.* tumor cell lines, comparable to its congener 10,11-dehydrocurvularin 29 (Fig. 2).^15,18^ Berkeleylactone A 28 displays strong antibacterial effects against Gram-positive bacteria, even though it is denoted as pro-drug of antibiotic A26771B 27. Here it is hypothesised that the sulfur side chain disrupts the γ-keto-α,β-unsaturated carboxyl, which is thought to be important for bioactivity of the macrolide antibiotic 27 (Fig. 2).^19,20^

In contrast, thiopleurotinic acid A 32, derived from the cytotoxic and antibacterial compound dihydropleurotinic acid 31 does not possess bioactivity (Fig. 2).^16^ Another case in which the addition of 2-hydroxy-3-mercaptopropanoic acid 22 is reported as a detoxification mechanism in fungi is antimicrobial compound Sch-642305 33. It is converted into a sulfur derivative 34 (Fig. 2) harboring the 2-hydroxy-3-mercaptopropanoic acid moiety in *Aspergillus niger*, losing its bioactivity in the process.^21^ Our results are also consistent with the hypothesis that addition of 2-hydroxy-3-mercaptopropanoic acid may be a self-resistance mechanism, as sporothioethers A 14 and B 15 display significantly reduced antifungal activity than the parent compound 8.

## Conflicts of interest

There are no conflicts to declare.

## Supplementary Material

RA-013-D3RA06542A-s001
