# Supplementary material for: Sporothioethers: deactivated alkyl citrates from the fungus Hypomontagnella monticulosa
Source: RSC Adv. 2023 Oct 11;13(42):29768–72. doi: 10.1039/d3ra06542a (PMC10565555; doi:10.1039/d3ra06542a)
Supplement: RA-013-D3RA06542A-s001 [file RA-013-D3RA06542A-s001.pdf]

Electronic Supplementary Material (ESI) for RSC Advances.
This journal is © The Royal Society of Chemistry 2023

Supporting Information

Sporothioethers: Deactivated alkyl citrates from the fungus *Hypomontagnella monticulosa*

Henrike Heinemann,[a] Kevin Becker,[a] Hedda Schrey,[b,c] Haoxuan Zeng,[b,c] Marc Stadler,[b,c] and Russell J. Cox[a]

a Institute for Organic Chemistry, Leibniz University Hannover
Schneiderberg 1B, 30167 Hannover (Germany), and
Centre for Biomolecular Drug Research (BMWZ)

b Department Microbial Drugs, Helmholtz Centre for Infection Research (HZI) and German Centre for Infection Research (DZIF), Partner Site Hannover/Braunschweig, Innenhoffenstraße 7, 30124 Braunschweig, Germany

c Institute of Microbiology, Technische Universität Braunschweig, Spielmannstraße 7, 38106 Braunschweig, Germany

Contents:

Experimental 2

Figure S1 LCMS analysis of purified sporothioether mixture (A 14 and B 15) 6

Figure S2 HRMS analysis of sporothioether mixture A 14 and B 15 6

Figure S3 LCMS analysis of chemically methylated sporothioether mixture 7

Figure S4 LCMS analysis of semisynthetic sporothioether mixture 8

Table S1: ^{1}H and ^{13}C NMR data of fungal 14 and 15 9

Figure S5−10: NMR spectra of fungal 14 and 15 10

Table S2: ^{1}H and ^{13}C NMR data of synthetic 14 and 15 16

Figure S11−15: NMR spectra of synthetic 14 and 15 17

Table S3: ^{1}H and ^{13}C NMR data of synthetic 24 and 25 22

Figure S16−20: NMR spectra of synthetic 24 and 25 23

Experimental

General Experimental Procedures

All chemicals and reagents were purchased from Sigma Aldrich, Carl Roth or Thermo Fischer Scientific. All solvents used for HPLC were analytical grade.

Nuclear Magnetic Resonsnace (NMR)

NMR data were recorded using either a Bruker Ultrashield 500 (^{1}H 500 MHz, ^{13}C 125 MHz), Bruker Ascend 600 (^{1}H 600 MHz, ^{13}C 150 MHz), Bruker Avance III 600 (^{1}H 600 MHz, ^{13}C 150 MHz) or Bruker Avance III 700 MH (^{1}H 700 MHz, ^{13}C 175 MHz) spectrometer. Chemical shifts are shown in parts per million (ppm) and referenced to solvents. The coupling constants J are given in Hz.

Analytical LCMS

LCMS data was obtained using a Waters LCMS system containing a 2767 autosampler, 2545 pump system, a Phenomenex Kinetex column (C18, 2.6 µm, 100, 4.6×100 mm) equipped with a Phenomenex Security Guard precolumn (Luna C5, 300 Å). Detection was done with Waters 2998 Diode Array detector at 210–600 nm; 2424 ELSD and SQD-2 mass detector operating simultaneously in ES^{+} and ES^{-} modes in a range of *m/z* 100–1000. The mobile phase was composed of HPLC-grade water mixed with 0.05 % formic acid (solvent A) and HPLC-grade acetonitrile with 0.045 % formic acid (B).

Fungal Strains, Fermentation, and Extraction

For compound isolation, *Hypomontagnella monticulosa* MUCL 54604 was grown in 4L scale (40× 500 mL-flasks with 100 mL medium each) in DPY medium (dextrin from potato starch 2%, polypeptone 1%, yeast extract 0.5%, KH_2PO_4 0.5%, $MgSO_4$ 0.05%) for 7 d at 28 °C with agitation at 130 rpm. Culture broth and mycelium were combined, shredded using a kitchen blender, and extracted twice with an equal amount of ethyl acetate. Combined organic layers were dried over $MgSO_4$ and solvent was removed under vacuum. Extracts were dissolved in methanol to a concentration of 20 mg/mL, filtered, and processed by preparative LCMS.

Preparative LCMS

Compound purification was done in analogy to the setup for analytical LCMS, but as column a Phenomenex Kinetex Axia column (C18, 5 µm, 100 Å, 21.2×250 mm) equipped with a Phenomenex Security Guard precolumn (Luna, C5, 300 Å) was used. A linear gradient from 10-90% solvent B was selected using a flow rate of 20 mL/min. Up to 900 µL of the crude extracts at 20 mg/mL were injected for each run. Waters Sample Manager was used to collect fractions by mass directed trigger. Combined fractions were dried, weighted and further analysed.

Chemical Derivatization of Sporothioethers Mixture

For methylation of the carboxyl group isolated sporothioether mixture **14**/**15** (3 mg) was dissolved in 1 mL MeOH and TMS-Diazomethane (2.0 M in diethyl ether, 3 eq.) was added dropwise to the stirred solution. After 12h the solvent was evaporated and the reaction product was further analyzed.

In vitro Synthesis of Sporothioether Derivate Mixture 30/ 31

For *in vitro* synthesis of a sporothioether derivate mixture 0.1 mg sporothriolide **8** was incubated with 2 eq. of 3-mercaptopropionic acid **29** in 50 mM phosphate buffer pH 7.5. After 1h the reaction was mixed 1:2 with ACN, filtered and analysed by LCMS.

Chemical Synthesis of Synthetic Sporothioether Mixtures 14/ 15 and 24/ 25 [1,2]

(*S*)-2-Hydroxy-3-(tritylthio)propanoic acid 20:

Triphenylmethanethiol (88 mg; 0.32 mmol) was dissolved in dry THF (1.2 mL) under a nitrogen atmosphere at to 0°C. Next, NaH (60% dispersion in mineral oil; 7.7 mg; 0.32 mmol) was added the solution was stirred at 0 °C for 15 min, before the potassium (*R*)-oxirane-2-carboxylate **18** (30 mg; 0.24 mmol) was added. The solution was gradually warmed to RT and stirred overnight. Next the solution was poured into H_2O (4 mL), extracted with Et_2O (3 × 1.6 mL) and the aqueous phase was acidified by 1 N HCl to pH 3-4, while the organic phase was discarded. The aqueous phase was extracted with EtOAc (3 × 1.6 mL). The combined organic layers were washed with brine, dried over anhydrous Na_2SO_4, and the remaining organic phase was concentrated to provide the product **20** as a yellow viscous oil (48 mg, 47 %).
R_f = 0.68 (MeOH/DCM = 1:1)

^{1}H NMR (400 MHz, DMSO-d_6): δ [ppm] = 2.33-2.35 (dd, *J* = 1.6, 6.2 Hz, 2H), 3.83 (dd, *J* = 5.6, 6.9 Hz, 1H), 5.55 (bs, 1H), 7.20−7.35 (m, 15H), 12.55 (bs, 1H);
^{13}C NMR (400 MHz, DMSO-d_6): δ [ppm] = 36.0, 65.8, 69.0, 126.7, 128.0, 129.1, 144.4, 173.8.
Mass spectrometry: HRMS (ESI$^-$): $C_{22}H_{19}O_3S$ ([M-H]$^-$ (calculated 363.1055, found 363.1060).
$[\alpha]_D^{25}$ = −38.0 (c = 0.53, $CHCl_3$)

(*S*)-2-Hydroxy-3-mercaptopropanoic acid 22:

20 (0.1 mmol; 35 mg) was dissolved in DCM (600 µL). To the reaction mixture triethylsilane (3 equiv.; 0.29 mmol; 46 µL) was added and the mixture was cooled to 0 °C with an ice bath. Next, trifluoroacetic acid (53.6 µL) was added dropwise. After completion, monitored by TLC, the reaction mixture was concentrated in vacuo. The crude product was washed 4 times with hexanes to remove starting material. After evaporation of solvent product **22** (9.36 mg, 80 %) was yielded as a white solid and directly used in subsequent sulfa-Michael addition.
R_f = 0.50 (MeOH/DCM = 1:1)

(3*R*, 15*S*), (3*S*, 15*S*)- Sporothioether mixture (14/ 15):[1,2]

Sporothriolide **8** (0.057 mmol, 16 mg) was dissolved in DCM (1.2 mL) followed by the addition of **22** (1.2 equiv.; 0.068 mmol; 8.3 mg) and Et_3N (5 equiv.; 0.29 mmol; 40 µL). The reaction mixture was stirred for 2 h at RT, concentrated, and separated by preparative reverse-phase HPLC.
NMR: see below
Mass spectrometry: HRMS (ESI$^-$): $C_{16}H_{23}O_7S$ ([M-H]$^-$ (calculated 359.1165, found 359.1163).

(*R*)-2-Hydroxy-3-(tritylthio)propanoic acid 21:

Triphenylmethanethiol (88 mg; 0.32 mmol) was dissolved in dry THF (1.2 mL) under a nitrogen atmosphere at to 0 °C. Next, NaH (60% dispersion in mineral oil; 7.9 mg; 0.32 mmol) was added the solution was stirred at 0 °C for 15 min, before the potassium (*S*)-oxirane-2-carboxylate **19** (30 mg; 0.24 mmol) was added. The solution was gradually warmed to RT and stirred overnight. Next the solution was poured into H_2O (16 mL), extracted with Et_2O (3 × 1.6 mL) and the aqueous phase was acidified by 1 N HCl to pH 3− 4, while the organic phase was discarded. The aqueous phase was extracted with EtOAc (3 × 1.6 mL). The combined organic layers were washed with brine, dried over anhydrous Na_2SO_4, and the remaining organic phase was concentrated to provide the product **21** as a yellow viscous oil (41 mg, 47 %)
R_f = 0.64 (MeOH/DCM = 1:1)
^{1}H NMR (400 MHz, DMSO-d_6): δ [ppm] = 2.33-2.35 (dd, *J* = 1.6, 6.4 Hz, 2H), 3.84 (m, 1H), 5.55 (bs, 1H), 7.19−7.36 (m, 15H), 12.5 (bs, 1H);
^{13}C NMR (400 MHz, DMSO-d_6): δ [ppm] = 36.0, 65.8, 69.0, 126.7, 128.0, 129.1, 144.4, 173.8.
Mass spectrometry: HRMS (ESI$^-$): $C_{22}H_{19}O_3S$ ([M-H]$^-$ (calculated 363.1055, found 363.1055).
$[\alpha]_D^{25}$ = 35.0 (c = 0.55, $CHCl_3$)

(*R*)-2-Hydroxy-3-mercaptopropanoic acid 23:

21 (0.12 mmol; 41 mg) was dissolved in DCM (650 µL). To the reaction mixture triethylsilane (3 equiv; 0.34 mmol; 55 µL) was added and the mixture was cooled to 0 °C with an ice bath. Next, trifluoroacetic acid (63.96 µL) was added dropwise. After completion, monitored by TLC, the reaction mixture was concentrated in vacuo. The crude product was washed 4 times with hexanes to remove starting material. After evaporation of solvent product **23** (9.4 mg, 67.1 %) was yielded as a white solid and directly used in subsequent sulfa Michael addition.
R_f = 0.48 (MeOH/DCM = 1:1)

(3*R*, 15*R*), (3*S*, 15*R*)- Sporothioether mixture (24/ 25):

Sporothriolide **8** (0.024 mmol, 5.7 mg) was dissolved in DCM (500 µL) and **23** (1.2 eq.; 0.029 mmol; 3.5 mg) and Et_3N (5 eq.; 0.12 mmol; 17 µL) were added. The reaction mixture was stirred for 2 h at RT. Next, it was concentrated and the crude product separated by preparative LCMS.
NMR: see below
Mass spectrometry: HRMS (ESI$^-$): $C_{22}H_{19}O_3S$ ([M-H]$^-$ (calculated 359.1165, found 359.1166).

Minimum Inhibitory Concentration (MIC) Assay

Compounds **14** and **15** mix., **24** and **25** mix., and **8** were tested against several bacterial and fungal strains by using a 96-well serial in Mueller-Hinton broth (MHB) media for bacteria and YMG media for filamentous fungi and yeasts as previously described.[3] The selected organisms represent a broad spectrum of pathogens of clinical interest, as well as sensitive indicator strains (Gram-positive bacteria: *Bacillus subtilis* DSM 10, *Staphylococcus aureus* DSM 346, *Mycobacterium smegmatis* ATCC 700084; Gram-negative bacteria: *Acinetobacter baumannii* DSM 30008, *Chromobacterium violaceum* DSM 30191, *Escherichia coli* DSM 1116, *Pseudomonas aeruginosa* PA 14; filamentous fungi: *Mucor hiemalis* DSM 2656; yeasts: *Candida albicans* DSM 1665, *Pichia anomala* DSM 6766, *Rhodotorula glutinis* DSM 10134, *Schizosaccharomyces pombe* DSM 70572). Compounds **14** and **15** mix., **24** and **25** mix., were tested against all of the organisms listed, but **8** was only tested against *Staphylococcus aureus* DSM 346 in this work, as this was not included in MIC assays reported previously. [3] Compounds **14** and **15** mix., **24** and **25** mix., and **8** were dissolved in MeOH (1 mg/mL), diluted to a final range of 66.6 to 0.52 µg/mL and incubated with the test organisms overnight. MeOH was used as negative control. Kanamycin (1.0 mg/mL; 2 µL [*M. smegmatis*]), vancomycin (10 mg/ml; 2 µL [MRSA]), gentamycin (1.0 mg/mL; 2 µL [*P. aeruginosa*]), ciprobay (2.54 mg/ml; 2 µL [*A. baumannii*]), nystatin (1.0 mg/mL; 20 µL [*S. pombe*, *P. anomala*, *M. hiemalis*, *C. albicans*, *R. glutinis*), and oxytetracycline (1.0 mg/mL; 2 µL [*C. violaceum*, *E. coli*, *S. aureus*] and 20 µL [*B. subtilis*]) were used as positive controls. The lowest inhibitory concentration of compounds **14** and **15** mix., **24** and **25** mix., and **8** (where no growth of the test organism was observed) was visually evaluated the next day.

Cytotoxicity Assay

KB-3-1 (ACC 158) cervix carcinoma as well as L929 (ACC 2) mouse fibroblasts were cultured under standard cell culture conditions (37 °C, 5% CO_2, 95% humidified atmosphere) as previously described.[4] The cells were maintained in high glucose DMEM containing 10% fetal bovine serum and 100 U/mL penicillin and streptomycin (Gibco, Thermo Fisher Scientific, Waltham, USA). Cell lines were serially passaged after trypsinization, using 0.05% trypsin / 0.02% EDTA solution. The cultures were routinely monitored for potential contamination, and only mycoplasma free cultures were used. The test compounds were investigated for their anti-proliferative effect on cell lines, using the 3-(4,5-dimethylthiazol-2-yl)-2,5-diphenyltetrazolium bromide (MTT) based cell viability assay. Cells were seeded in 96 well microtiter plates (Sarstedt, Germany) with a cell density of 0.05 * 10^6 cells per mL and 100 µL per well and were incubated for 24 h. A dilution series of test compounds was added to the wells, ranging in eleven steps from 37 µg/mL – 0.63 ng/mL, and equal amounts of DMSO or methanol were added as solvent controls. Treated cells were incubated for a further 72 h. Then, 12.5 µL per well of MTT solution (0.05% in PBS) were added, followed by another 2 h of incubation. The plates were centrifuged, the medium was discarded and 25 µL per well of SDS solution in DMSO (10% SDS, 0.6% AcOH in DMSO) were added to dissolve the formazan. The plates were incubated for another hour. Absorbances at 570 nm and 630 nm were measured with a plate reader (Tecan, Männedorf, canton of Zürich, Switzerland). Background absorbance (630 nm) was subtracted from the formazan signal (570 nm). The resulting absorbance is directly proportional to the amount of viable cells. The control was normalized to 100% viable cells, and the viability of cells treated with the test compounds was calculated accordingly. IC_{50} values were calculated based on a sigmoidal fit model using GraphPad Prism. Means and SD were calculated from at least four independent experiments.

Biofilm Assays

Biofilm inhibition

The stock of ***S. aureus* DSM 1104** was taken from –20 °C and incubated overnight in 25 mL CASO (casein-peptone soymeal-peptone) medium in a 250 mL flask at 37 °C shaking at 100 rpm. The OD_{600} of the culture solution was adjusted to the turbidity of a 0.001 McFarland standard using spectrophotometer (Nanodrop 2000c, Thermo Fisher Scientific, Waltham, USA). Afterwards, 150 µL of CASO medium, supplemented with 4% glucose, was added with serially diluted compounds (250–2 µg/mL) and incubated in a 96-well microtiter plate (tissue culture ref.no 92196, TPP, Switzerland) at 37 °C for 18 h. The activity against biofilm was evaluated via staining with 0.1% crystal violet (CV, Thermo Fisher, Waltham, MA, USA) following the established protocol.[4] Afterwards, the supernatant was removed, the biofilm was stained at room temperature with crystal violet for 15 min and washed three times with PBS (phosphate-buffered saline) buffer. The biofilm was dissolved with 95% ethanol, and the absorbance was quantified with a plate reader (Synergy 2, BioTek, Santa Clara, USA) at 530 nm. Methanol (2.5%) served as a negative control and microporenic acid A[4] (250–2 µg/mL) as a positive control. All experiments were run in duplicates with two repetitions. SD of two repeats with duplicates each were 10% or less.

The fungal pathogen ***C. albicans*** **DSM 11225** was taken from a stock at −20 °C in 25 mL YPD medium in a 250 mL flask at 30 °C at 100 rpm for 18 h. Turbidity of the preculture was measured at 280 nm using a spectrophotometer and diluted to match the turbidity of a 0.05 McFarland standard in RPMI 1640 medium (Gibco, Thermo Fisher Scientific). Subsequently, 150 µL of the fungal dispersion were added into each well of a 96-well microtiter plate (Falcon no. 351172, Thermo Fisher Scientific) and further incubated for 90 min, following the established protocol.[5] The supernatant was discarded and the plate was rinsed one time by using PBS buffer. Afterwards, compounds were serially diluted in 150 µL in fresh medium to concentrations of 250 – 2 µg/mL. Methanol (2.5%) was used as a solvent control and farnesol (Sigma Aldrich, St. Louis, MO, USA) as positive control (250 – 2 µg/mL). The plate was further incubated at 37 °C at 150 rpm for 24 h. The supernatant was discarded and biofilms were washed with PBS, stained by adding 150 µL CV solution (0.1%) and incubated (room temperature, 25 min). Afterwards, the plate was washed twice with PBS buffer. 150 µL ethanol (95%) were applied to dissolve the biofilm. The absorbance was measured by a plate reader at 610 nm. SD of two repeats with duplicates each were 10% or less.

Dispersion of preformed biofilms

S. aureus **DSM 1104** was taken from −20 °C stock and precultured in 25 mL CASO medium at 37 °C in 250 mL a flask at 100 rpm for 20 h. The precultured suspension of *S. aureus* was adjusted so that their OD_{600} matched the turbidity of a 0.001 McFarland standard. *S. aureus* was incubated in 96-well tissue plates (tissue culture ref.no 92196) for 24 h at 150 rpm in 150 mL CASO medium, supplemented with 4% glucose. The supernatant was removed from the wells and 150 µL of the respective media (fresh) was added to the wells, together with the serially diluted compounds (250–2 µg/mL), following the established protocol.[4] Methanol (2.5%) and microporenic acid A (250–2 µg/mL) were used as solvent and positive controls, respectively. The plate was incubated for a further 24 h at 37 °C. Staining of the biofilms and the controls was carried out as described above. SD of two repeats with duplicates each were 15% or less.

Figure S1 LCMS analysis of purified sporothioether mixture (A 14 and B 15)

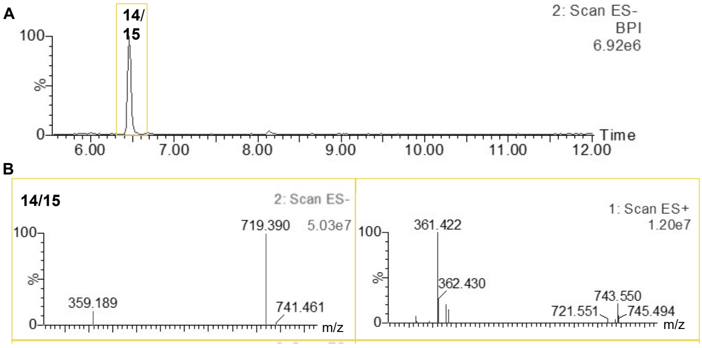

Fig. S1 LCMS analysis of sporothioether mixture. **A**, Linear gradient from 10 % to 90 % ACN; **B**, ES^+ and ES^- spectra of sporothioether mixture **14** and **15**.

Figure S2 HRMS analysis of sporothioether mixture A 14 and B 15

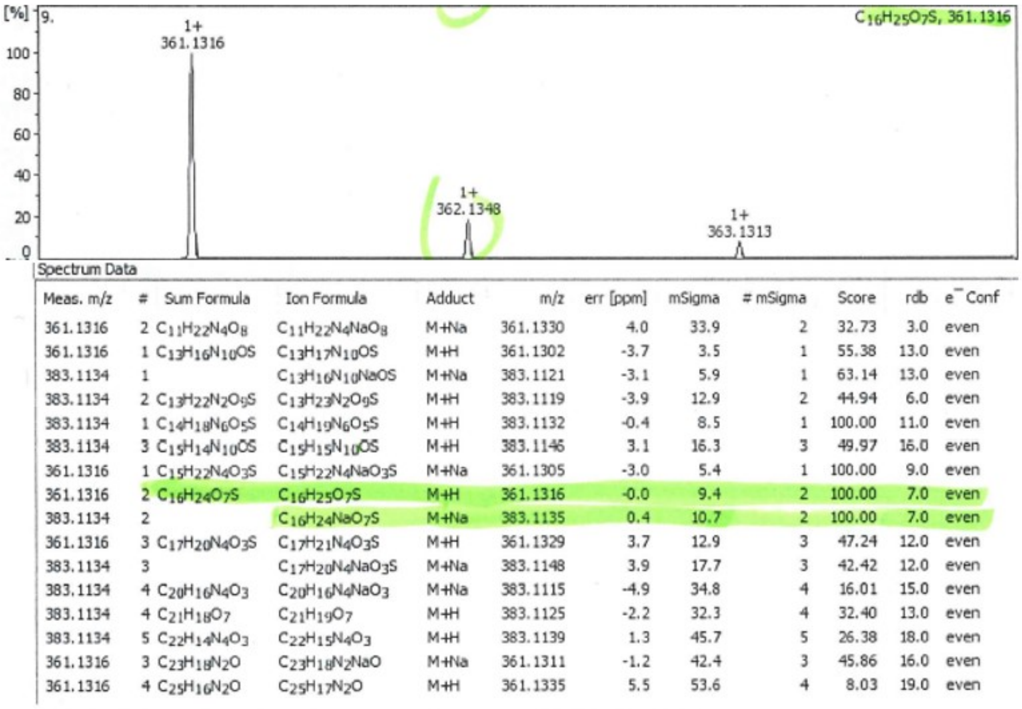

Spectrum Data

Meas. m/z	#	Sum Formula	Ion Formula	Adduct	m/z	err [ppm]	mSigma	# mSigma	Score	rdb	e⁻ Conf
361.1316	2	$C_{11}H_{22}N_4O_8$	$C_{11}H_{22}N_4NaO_8$	M+Na	361.1330	4.0	33.9	2	32.73	3.0	even
361.1316	1	$C_{13}H_{16}N_{10}OS$	$C_{13}H_{17}N_{10}OS$	M+H	361.1302	-3.7	3.5	1	55.38	13.0	even
383.1134	1		$C_{13}H_{16}N_{10}NaOS$	M+Na	383.1121	-3.1	5.9	1	63.14	13.0	even
383.1134	2	$C_{13}H_{22}N_2O_9S$	$C_{13}H_{23}N_2O_9S$	M+H	383.1119	-3.9	12.9	2	44.94	6.0	even
383.1134	1	$C_{14}H_{18}N_6O_5S$	$C_{14}H_{19}N_6O_5S$	M+H	383.1132	-0.4	8.5	1	100.00	11.0	even
383.1134	3	$C_{15}H_{14}N_{10}OS$	$C_{15}H_{15}N_{10}OS$	M+H	383.1146	3.1	16.3	3	49.97	16.0	even
361.1316	1	$C_{15}H_{22}N_4O_3S$	$C_{15}H_{22}N_4NaO_3S$	M+Na	361.1305	-3.0	5.4	1	100.00	9.0	even
361.1316	2	$C_{16}H_{24}O_7S$	$C_{16}H_{25}O_7S$	M+H	361.1316	-0.0	9.4	2	100.00	7.0	even
383.1134	2		$C_{16}H_{24}NaO_7S$	M+Na	383.1135	0.4	10.7	2	100.00	7.0	even
361.1316	3	$C_{17}H_{20}N_4O_3S$	$C_{17}H_{21}N_4O_3S$	M+H	361.1329	3.7	12.9	3	47.24	12.0	even
383.1134	3		$C_{17}H_{20}N_4NaO_3S$	M+Na	383.1148	3.9	17.7	3	42.42	12.0	even
383.1134	4	$C_{20}H_{16}N_4O_3$	$C_{20}H_{16}N_4NaO_3$	M+Na	383.1115	-4.9	34.8	4	16.01	15.0	even
383.1134	4	$C_{21}H_{18}O_7$	$C_{21}H_{19}O_7$	M+H	383.1125	-2.2	32.3	4	32.40	13.0	even
383.1134	5	$C_{22}H_{14}N_4O_3$	$C_{22}H_{15}N_4O_3$	M+H	383.1139	1.3	45.7	5	26.38	18.0	even
361.1316	3	$C_{23}H_{18}N_2O$	$C_{23}H_{18}N_2NaO$	M+Na	361.1311	-1.2	42.4	3	45.86	16.0	even
361.1316	4	$C_{25}H_{16}N_2O$	$C_{25}H_{17}N_2O$	M+H	361.1335	5.5	53.6	4	8.03	19.0	even

Fig. S2 HRMS analysis of sporothioether mixture A **14** and B **15**.

Figure S3 LCMS analysis of chemically methylated sporothioether mixture

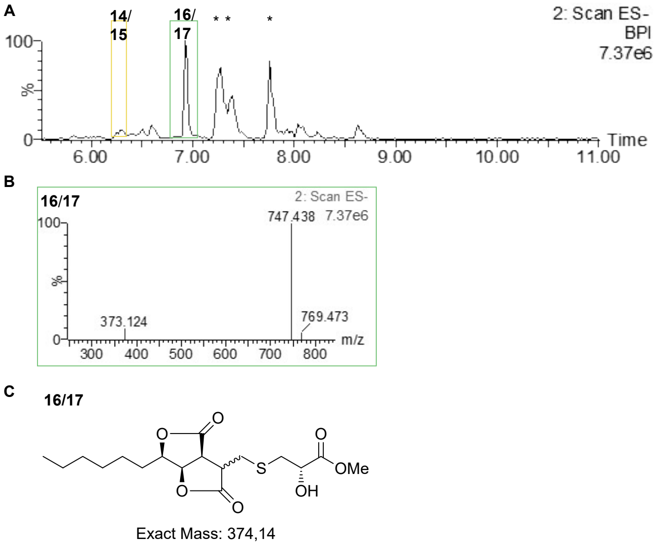

Fig. S3 LCMS analysis of methylation reaction of sporothioether mixture (A **14** and B **15**) towards **16** and **17**. **A**, Linear gradient from 10- 90% ACN; **B**, ES^- spectrum of methylated sporothioether mixture; **C**, Chemical structure of **16**/**17**. *= unrelated compounds.

Figure S4 LCMS analysis of semisynthetic sporothioether mixture

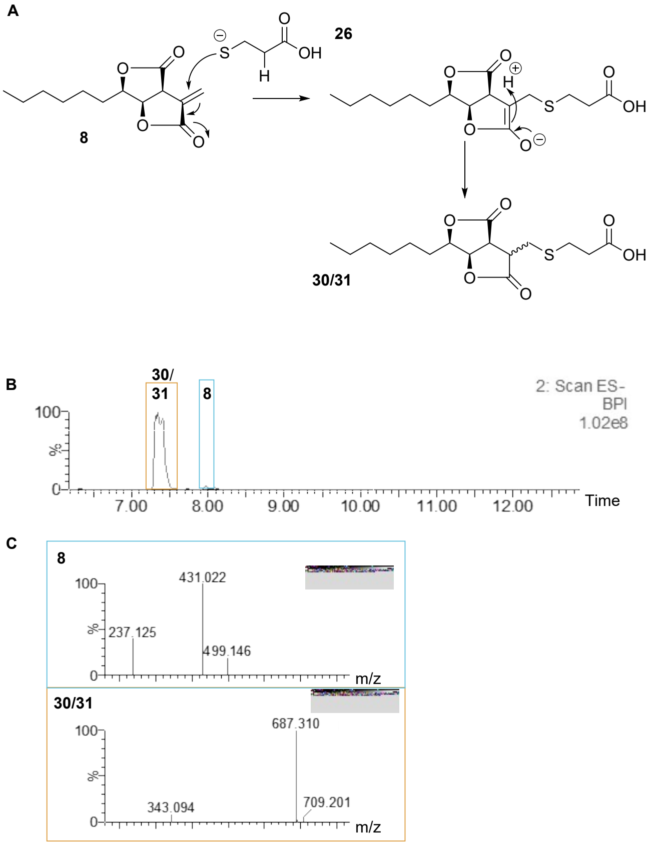

Fig. S4 LCMS analysis of semisynthetic sporothioether mixture. **A**, Proposed mechanism for synthesis of semisynthetic sporothioether mixture **30** and **31**; **B**, Linear gradient from 10 % to 90 % CAN; **C**, ES^{-} spectra of sporothioether mixture (**30** and **31**) and sporothriolide **8**.

Table S1: ^{1}H and ^{13}C NMR data of fungal 14 and 15

Table S1. ^{1}H and ^{13}C NMR spectroscopic data of sporothioether A and B (**14** and **15**). Chemical shifts are expressed in ppm. Samples were measured in MeOD (^{1}H: 700 MHz, ^{13}C: 175 MHz).

**position**	**14** (sporothioether A)		**15** (sporothioether B)	
	^{13}C	^{1}H	^{13}C	^{1}H
1	174.6, C	-	177.5, C	-
2	45.0, CH	3.84, dd (9.2, 5.5)	47.7, CH	3.79, dd (6.2, 1.5)
3	44.8, CH	3.66, td (9.2, 6.4)	45.6, CH	3.17, ddd (8.9, 4.5, 1.5)
4	176.1, C	-	177.0, C	-
5	80.4, CH	5.14, dd (5.5, 3.7)	81.3, CH	5.28, dd (6.2, 3.9)
6	82.5, CH	4.63, ddd (8.0, 6.5, 3.7)	83.6, CH	4.71, ddd (7.9, 6.5, 4.1)
7	29.8, CH_2	1.81, m	29.8, CH_2	1.81, m
8	26.3, CH_2	1.50, m	26.3, CH_2	1.5, m
9	30.0, CH_2	1.41, m	30.0, CH_2	1.41, m
10	32.7, CH_2	1.34, br d (4.3)	32.7, CH_2	1.34, br d (4.3)
11	23.5, CH_2	1.35, m	23.5, CH_2	1.35, m
12	14.2, CH_3	0.93, m	14.2, CH_3	0.93, m
13	29.8, CH_2	3.06, dd (8.9, 1.7)	34.3, CH_2	3.24, dd (13.5 4.5)<br>2.96, dd (13.5, 8.8)
14	38.4, CH_2	3.00, m	37.1, CH_2	3.08, dd (10.2, 3.9)<br>2.88, dd (14.1, 6.3)
15	72.3, CH	4.36, dd (5.9, 4.0)	72.3, CH	4.34, br dd (6.3, 3.9)
16	176.4, C	-	176.3, C	-

Figures S5−10: NMR spectra of fungal 14 and 15

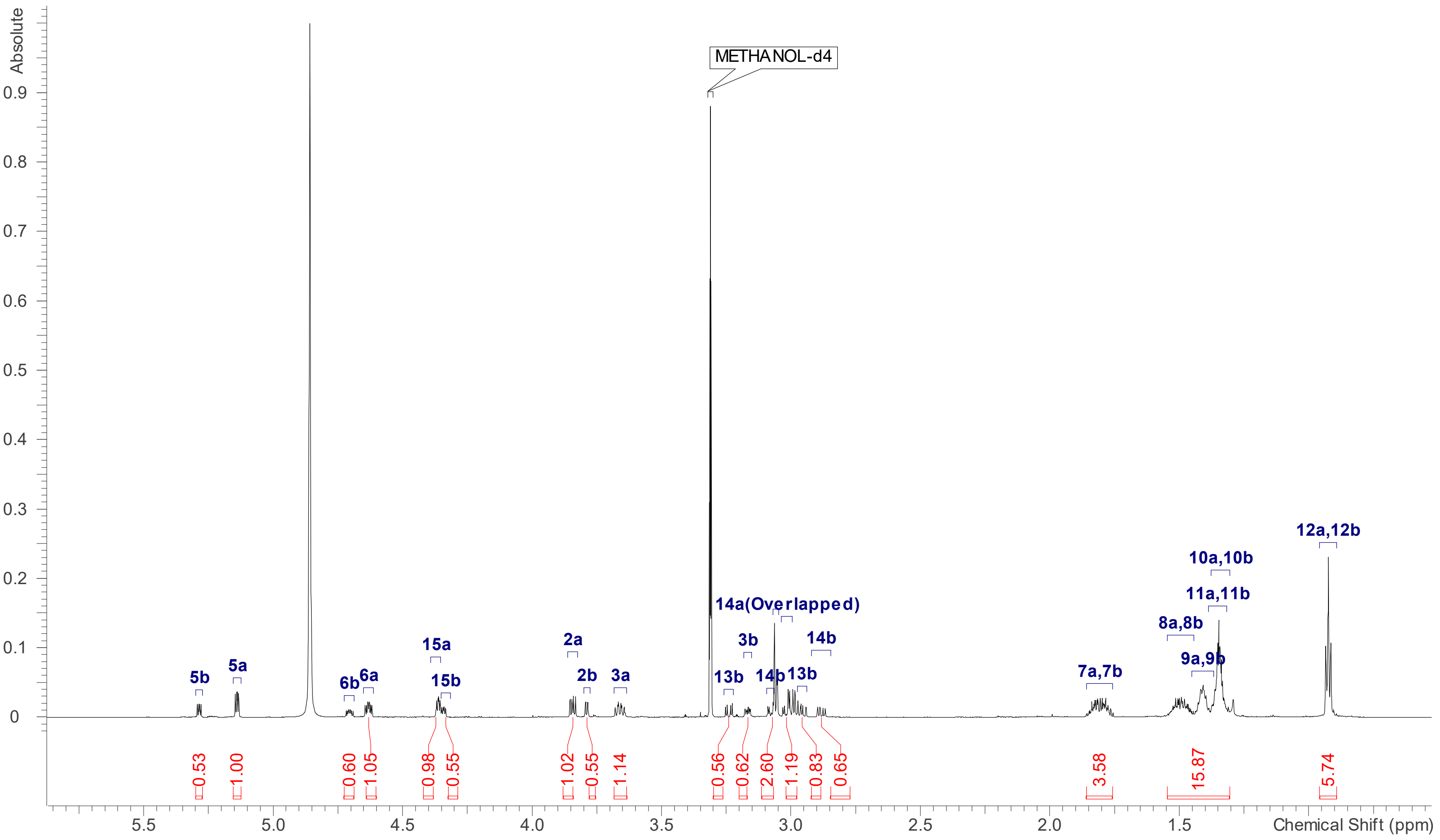

Fig. S5 ^{1}H NMR spectrum (700 MHz, MeOD) of fungal **14** and **15**.

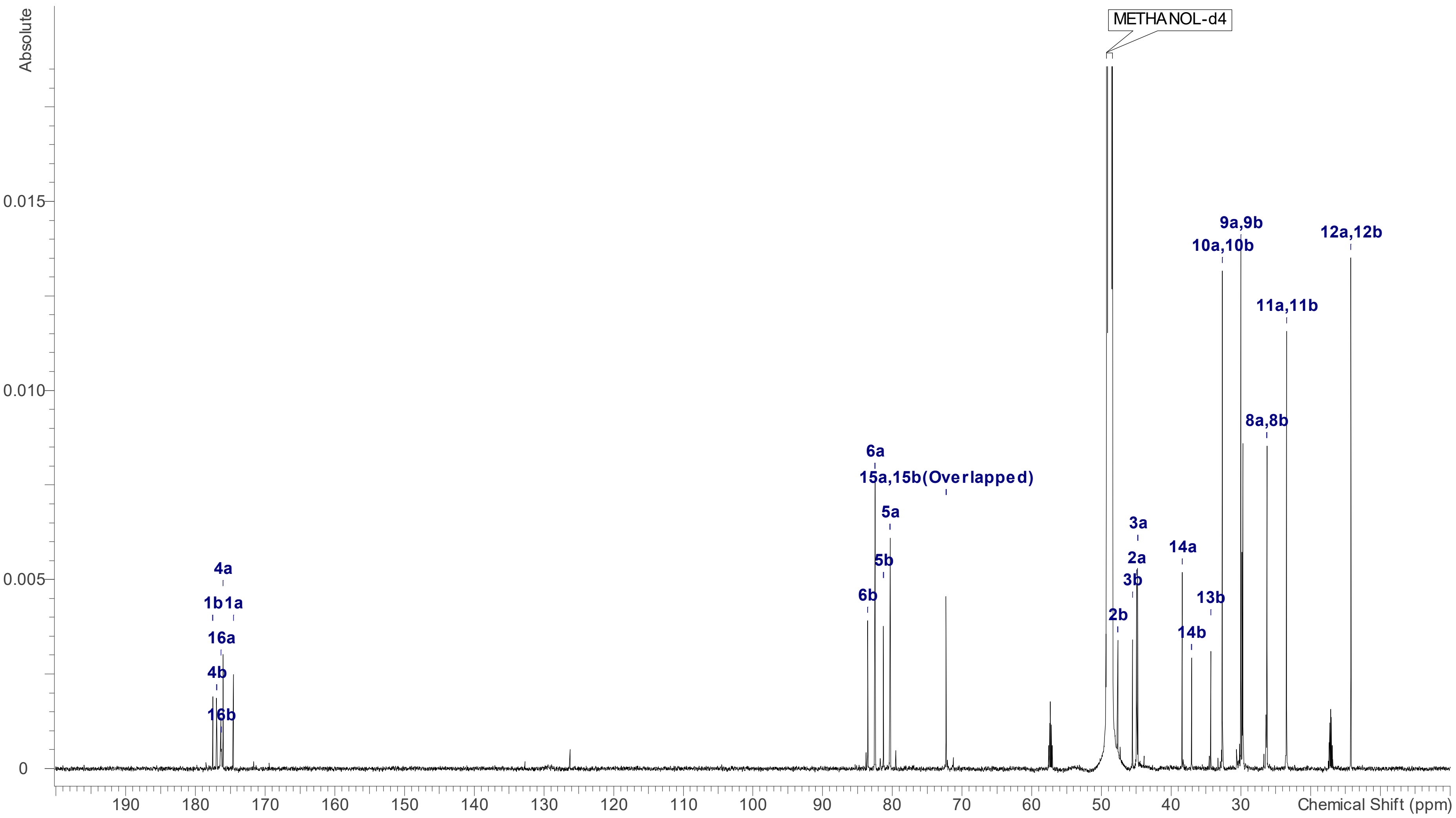

Fig. S6 ^{13}C NMR spectrum (175 MHz, MeOD) of fungal **14** and **15**.

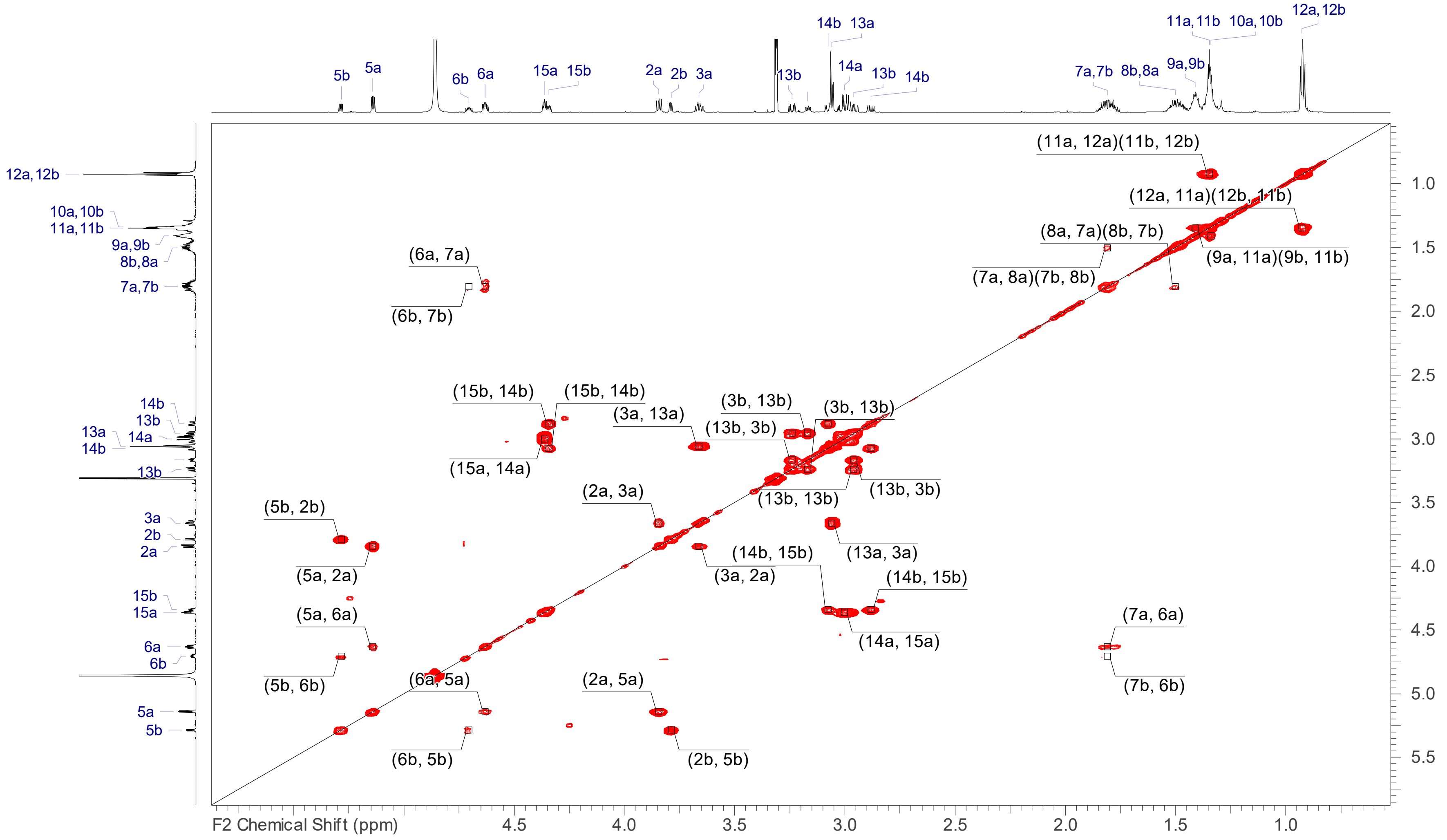

Fig. S7 $^{1}H/^{1}H$ COSY spectrum (700 MHz, MeOD) of fungal **14** and **15**.

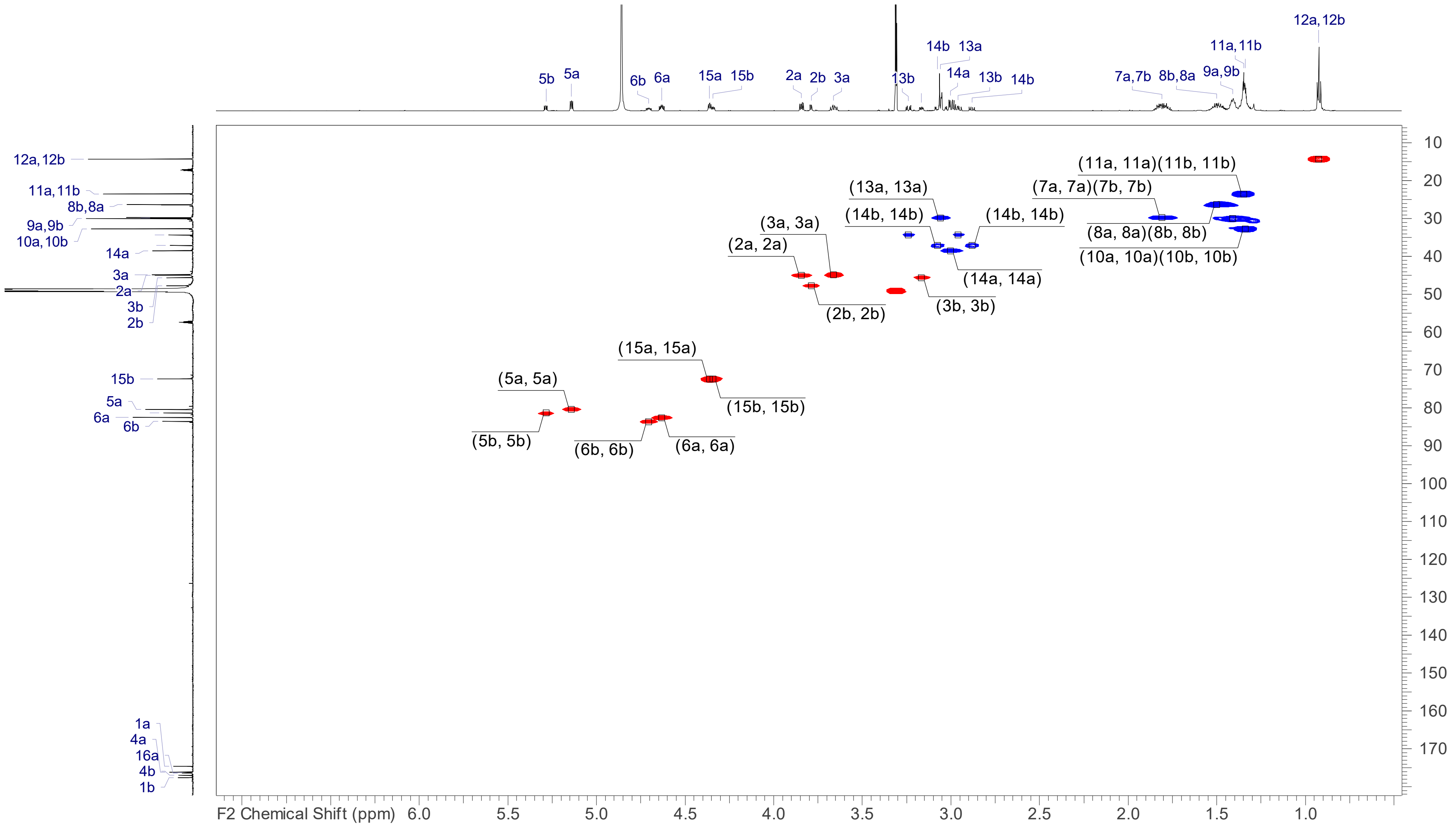

Fig. S8 $^{1}H/^{13}C$ HSQC spectrum (700 MHz, MeOD) of fungal **14** and **15**.

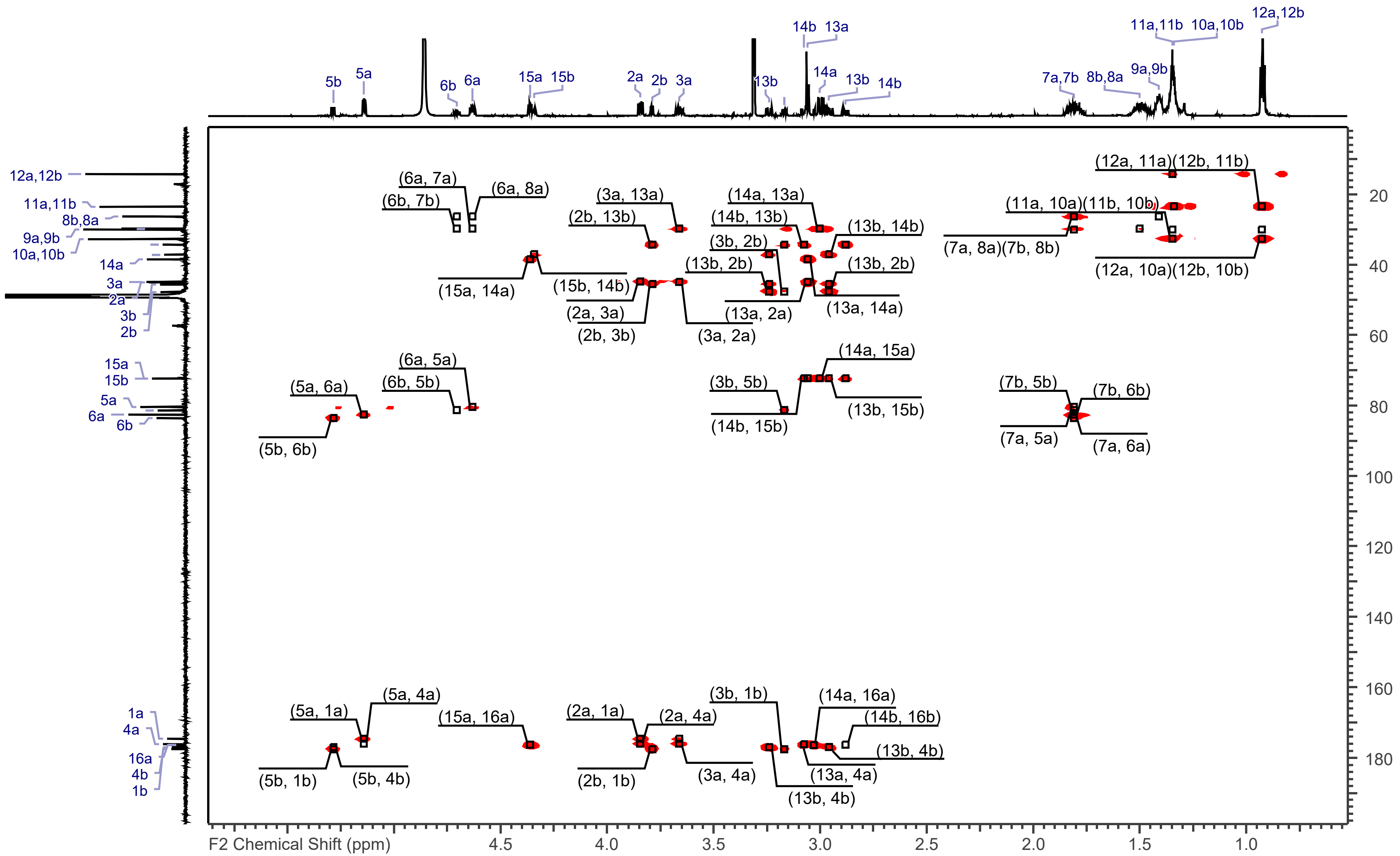

Fig. S9 $^{1}H/^{13}C$ HMBC spectrum (700 MHz, MeOD) of fungal **14** and **15**.

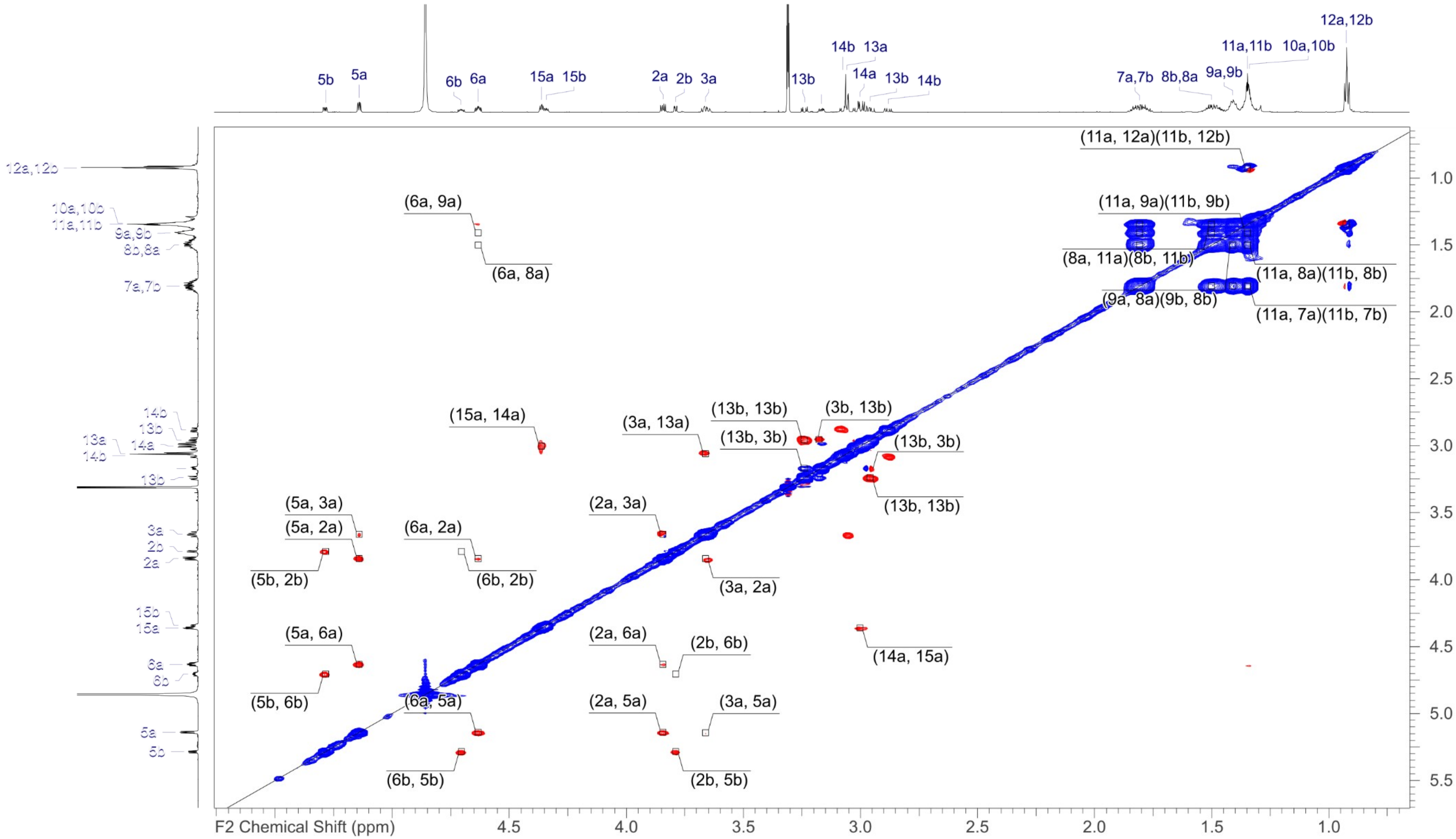

Fig. S10 ^{1}H/^{1}H ROESY spectrum (700 MHz, MeOD) of fungal **14** and **15**.

Table S2: ^{1}H and ^{13}C NMR data of synthetic 14 and 15

Table S2. ^{1}H and ^{13}C NMR spectroscopic data of synthetic sporothioether A and B (**14** and **15**). Chemical shifts are expressed in ppm. Samples were measured in MeOD (^{1}H: 600 MHz, ^{13}C: 150 MHz).

**position**	**3*R*, 15*S* sporothioether A 14 (minor compound)**				**3*S*, 15*S* sporothioether B 15 (major compound)**			
	^{13}C	^{1}H	HMBC	COSY	^{13}C	^{1}H	HMBC	COSY
1	174.7, C	-	-	-	177.7, C	-	-	-
2	45.1, CH	3.84, dd (9.3, 5.3)	4, 1, 3	5, 3	47.8, CH	3.79, dd (6.2, 1.6)	13, 3, 4	5, 3
3	45.0, CH	3.66, m	13, 2, 4, 1	2, 13	45.7, CH	3.17, ddd (8.8, 4.5, 1.6)	13,5, 1, 4	13, 2
4	176.2, C	-	-	-	177.1, C	-	-	-
5	80.5, CH	5.14, dd (5.3, 3.6)	4	2, 6	81.5, CH	5.3, dd (6.2, 4.0)	6, 4	2, 6
6	82.6, CH	4.63 ddd (7.9, 6.4, 3.6)	5	7, 5	83.7, CH	4.71, ddd (7.9, 6.4, 4.0)	8, 5	7, 5
7	29.9, CH_2	1.81, m	8, 6	8, 6	29.9, CH_2	1.81, m	8, 6	8, 6
8	26.4, CH_2	1.50, m	9, 7	-	26.5, CH_2	1.50, m	9, 7	-
9	30.1, CH_2	1.41, m	10, 8	-	30.1, CH_2	1.41, m	10, 8	-
10	32.8, CH_2	1.35, m	11, 9	11, 9	32.8, CH_2	1.35, m	11	11
11	23.6, CH_2	1.35, m	12, 10	12, 10	23.6, CH_2	1.35, m	12, 10	12, 10
12	14.4, CH_3	0.92, t	11, 10	11, 10	14.4, CH_3	0.92, t	11, 10	11, 10
13	28.5, CH_2	3.05, m	3, 2, 4	3, 15, 14	34.5, CH_2	3.24, dd (13.5, 4.5)<br>2.96, dd (13.5, 8.8)	4, 2, 3	3
14	38.6, CH_2	3.0, m	-	15	37.3, CH_2	2.89, dd (13.9, 6.3)<br>3.09, overlapping dd	13, 15, 14	15, 14
15	72.5, CH	4.36, m	14, 16	14	72.5, CH	4.34, dd (6.5, 3.9)	14, 16	14
16	176.5, C	-	-	-	176.5, C	-	-	-

Figure S11−15: NMR spectra of synthetic 14 and 15

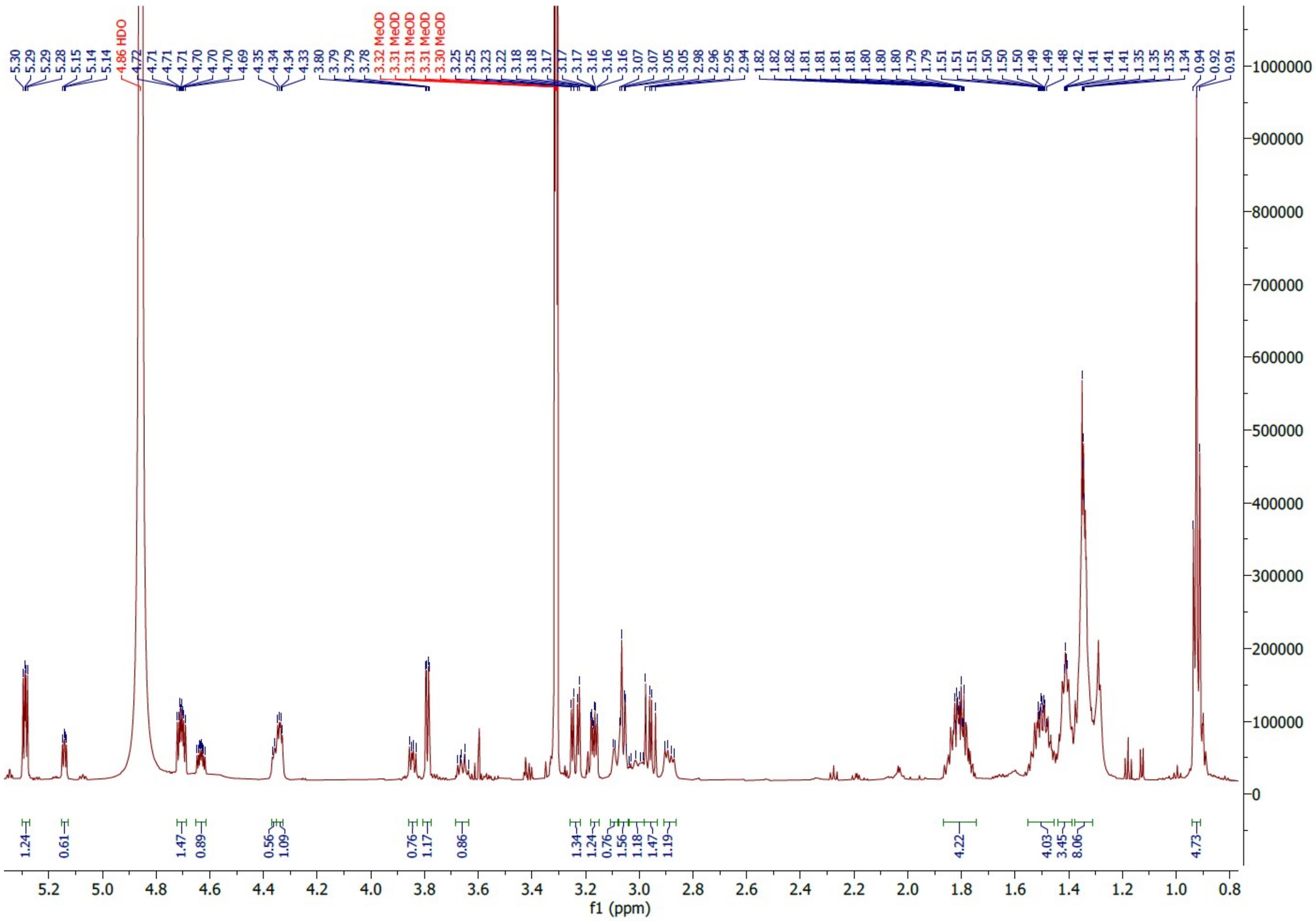

Fig. S11 ^{1}H NMR spectrum (600 MHz, MeOD) of synthetic **14** and **15**.

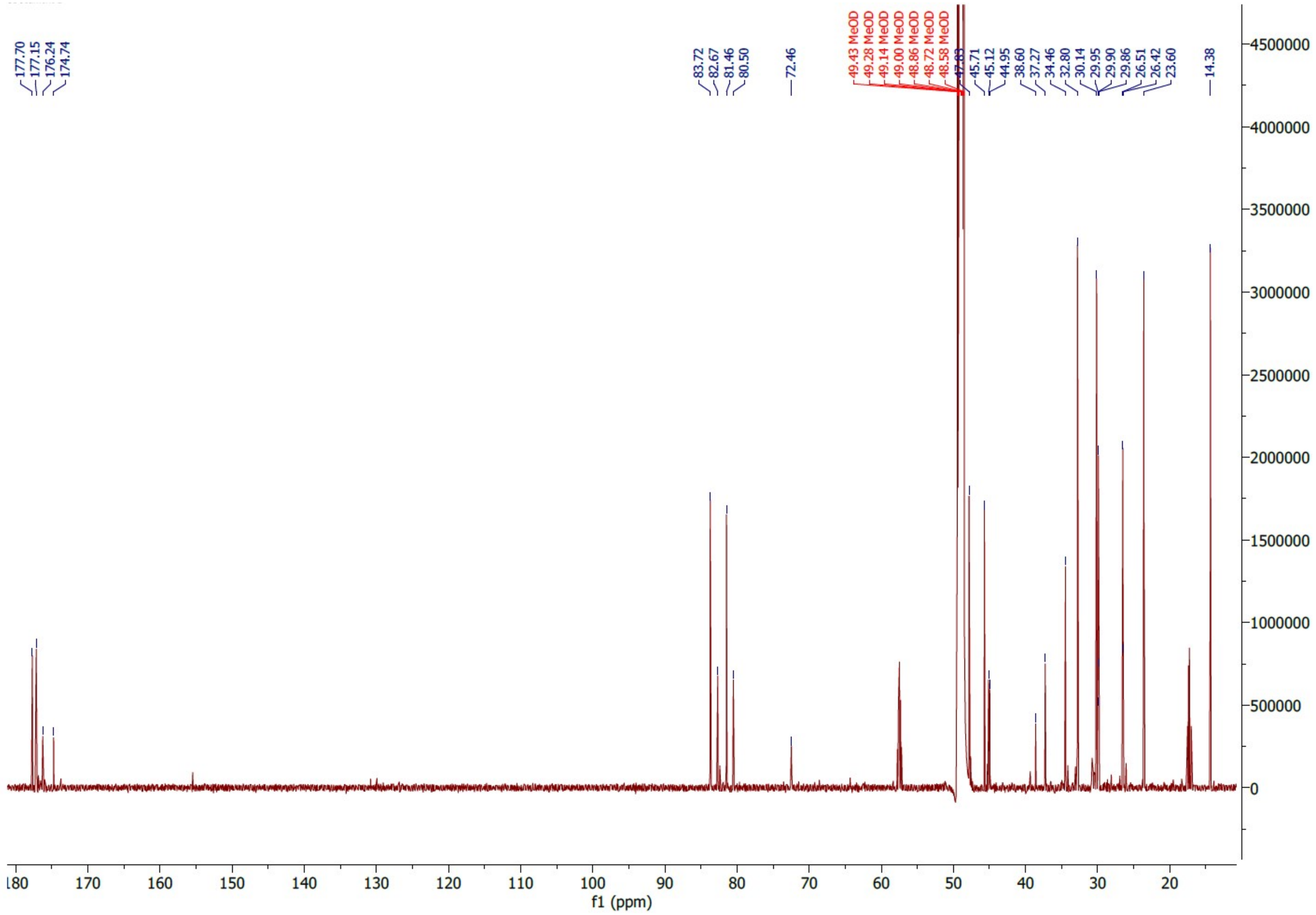

Fig. S12 ^{13}C NMR spectrum (150 MHz, MeOD) of synthetic **14** and **15**.

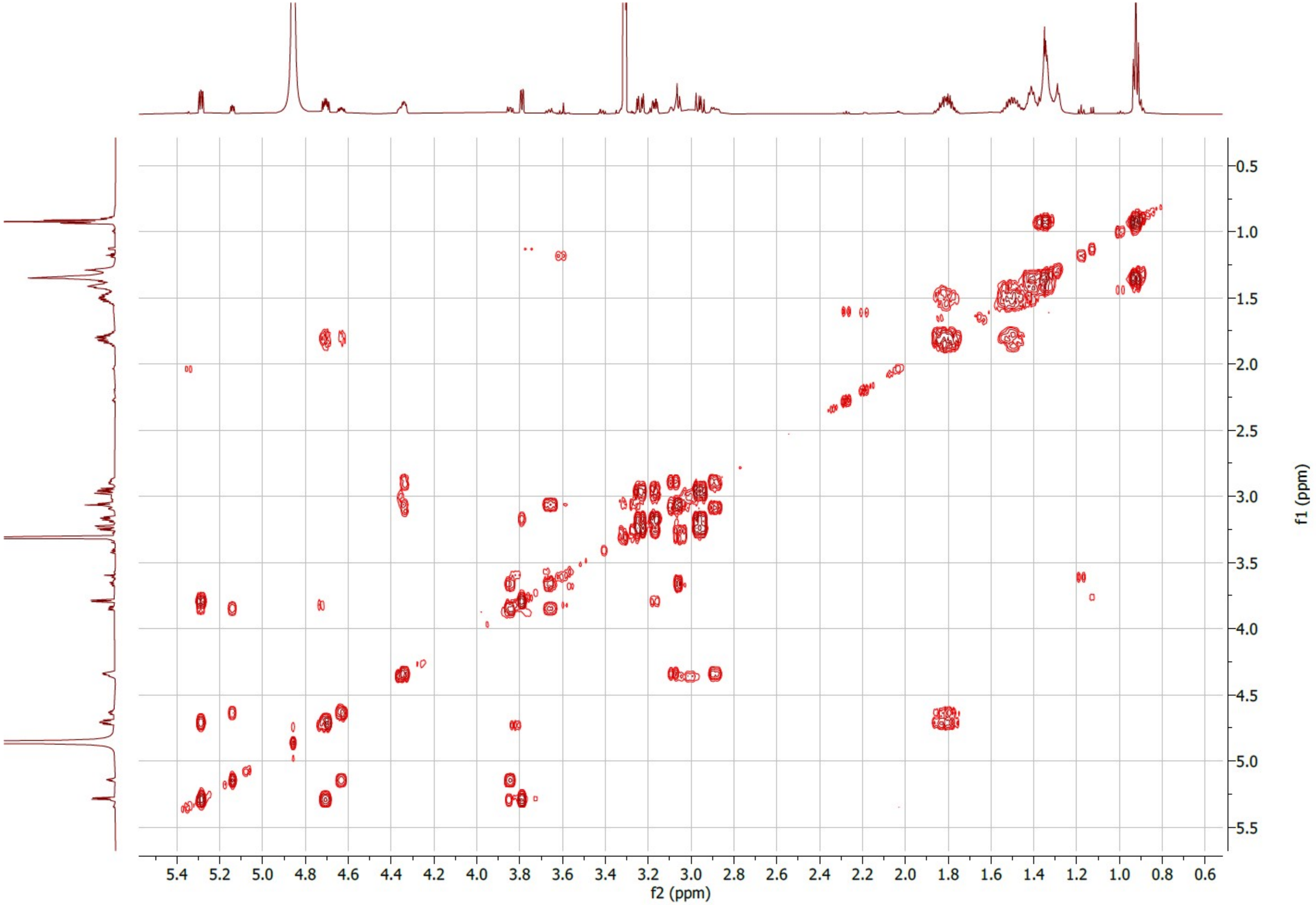

Fig. S 13 ^{1}H/^{1}H COSY spectrum (600 MHz, MeOD) of synthetic **14** and **15**.

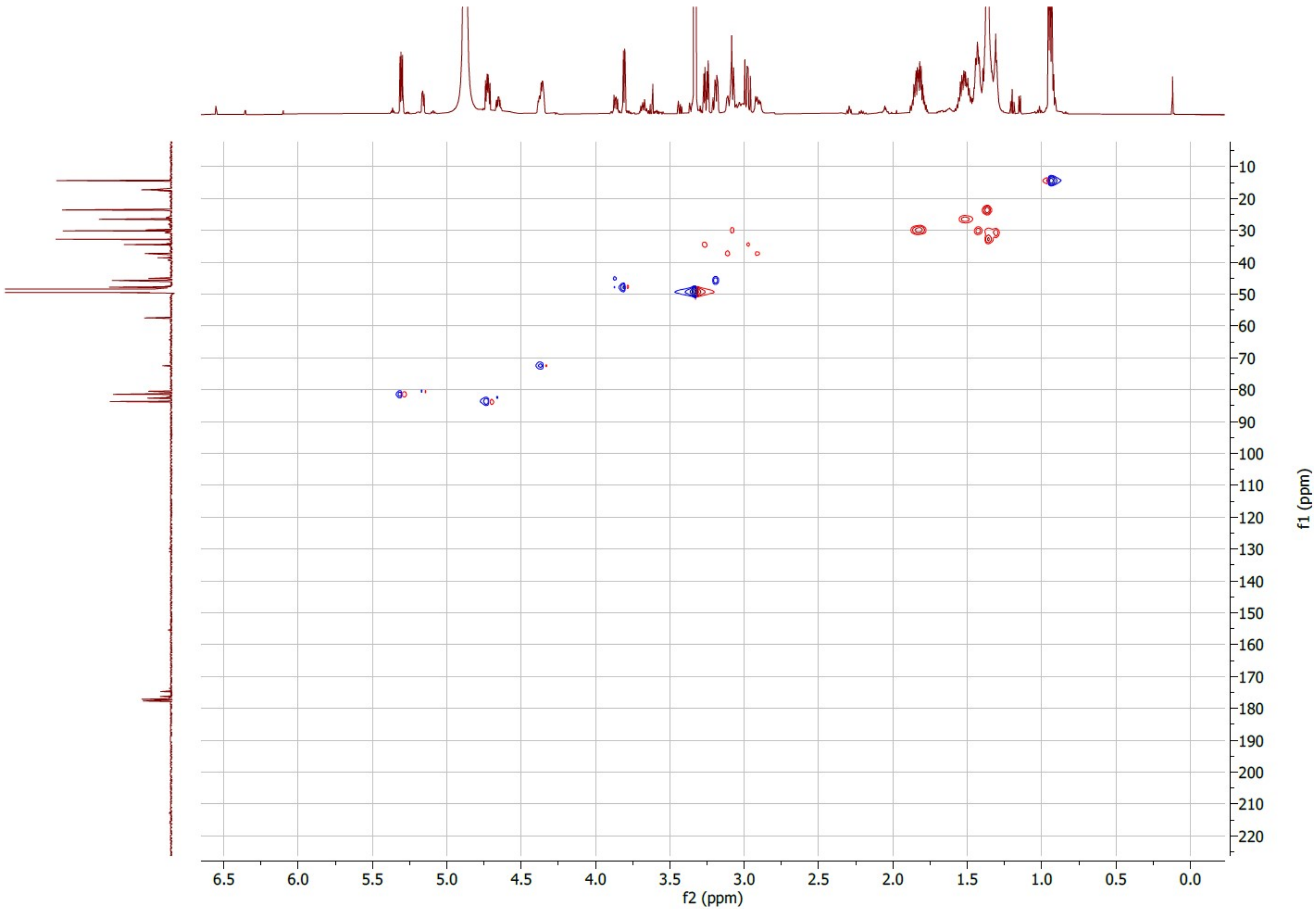

Fig. S14 $^{1}H/^{13}C$ HSQC spectrum (600 MHz, MeOD) of synthetic **14** and **15**.

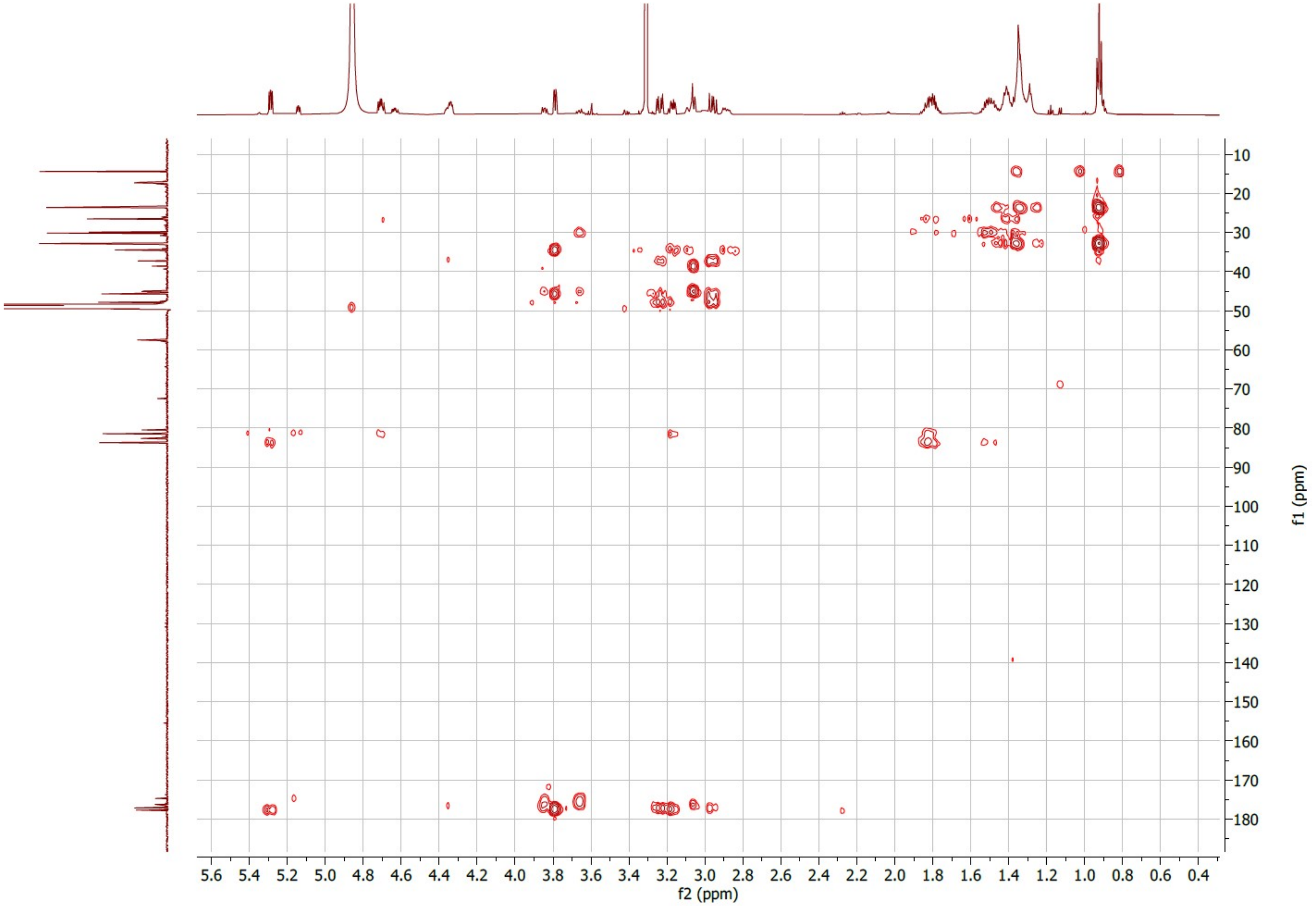

Fig. S 15 $^{1}H/^{13}C$ HMBC spectrum (600 MHz, MeOD) of synthetic **14** and **15.**

Table S3: ^{1}H and ^{13}C NMR data of synthetic 24 and 25

Table S3. ^{1}H and ^{13}C NMR spectroscopic data of synthetic sporothioether C and D (**24** and **25**). Chemical shifts are expressed in ppm. Samples were measured in MeOD (^{1}H: 500 MHz, ^{13}C: 125 MHz).

**position**	**3*R*, 15*R* sporothioether C 24 (minor compound)**				**3*S*, 15*R* sporothioether D 25 (major compound)**			
	^{13}C	^{1}H	HMBC	COSY	^{13}C	^{1}H	HMBC	COSY
1	174.7	-	-	-	177.8	-	-	-
2	45.1, CH	3.84, dd (9.3, 5.5)	3, 1	3, 5	47.8, CH	3.82, dd (6.2, 1.5)	13, 3, 4, 1	3, 5
3	44.7, CH	3.56, td (9.3, 9.1, 5.9)	4, 2, 13	13, 2	45.8, CH	3.19, ddd (8.3, 4.5, 1.5)	4, 13, 5, 2	13, 2
4	176.2, C	-	-	-	177.2	-	-	-
5	80.5, CH	5.14, dd (5.5, 3.6)	1, 6	2, 6	81.5, CH	5.28, dd (6.2, 4.0)	1, 6	2, 6
6	82.6, CH	4.63, ddd (7.8, 6.5, 3.6)	8, 7, 5	7, 5	83.7, CH	4.71, ddd (7.8, 6.6, 4.0)	8, 7, 5	7, 5
7	29.8, CH_2	1.82, m	8, 9, 6	8, 6	29.9, CH_2	1.82, m	8, 9, 6	8, 6
8	26.4, CH_2	1.50, m	9, 10, 7	7	26.5, CH_2	1.50, m	9, 10, 7	7
9	30.1, CH_2	1.41, m	10, 8, 11	-	30.2, CH_2	1.41, m	10, 8, 11	-
10	32.8, CH_2	1.35, m	11, 9	-	32.8, CH_2	1.35, m	11, 9	-
11	23.6, CH_2	1.35, m	12, 10	12	23.6, CH_2	1.35, m	12, 10	12
12	14.4, CH_3	0.92, m	11, 12	11	14.4, CH_2	0.92, m	11, 12	11
13	29.6, CH_2	3.1, m	4, 2, 14	14, 3, 15	34.7, CH_2	3.24, dd (13.1, 4.5)<br>2.96, dd (13.1, 8.3)	14, 3, 2, 15, 4	13, 14, 15, 3
14	38.4, CH_2	3.11, m<br>2.89, dd, (14.2, 6.9)	13, 15	13, 15	37.5, CH_2	2.98, m	3, 15, 13	13, 15
15	72.4, CH	4.32, dd (6.9, 3.8)	14, 16	14	72.5, CH	4.35, dd (6.2, 4.0)	16, 14	14
16	176.5, C	-	-	-	176.5, C	-	-	-

Figure S16−20: NMR spectra of synthetic 24 and 25

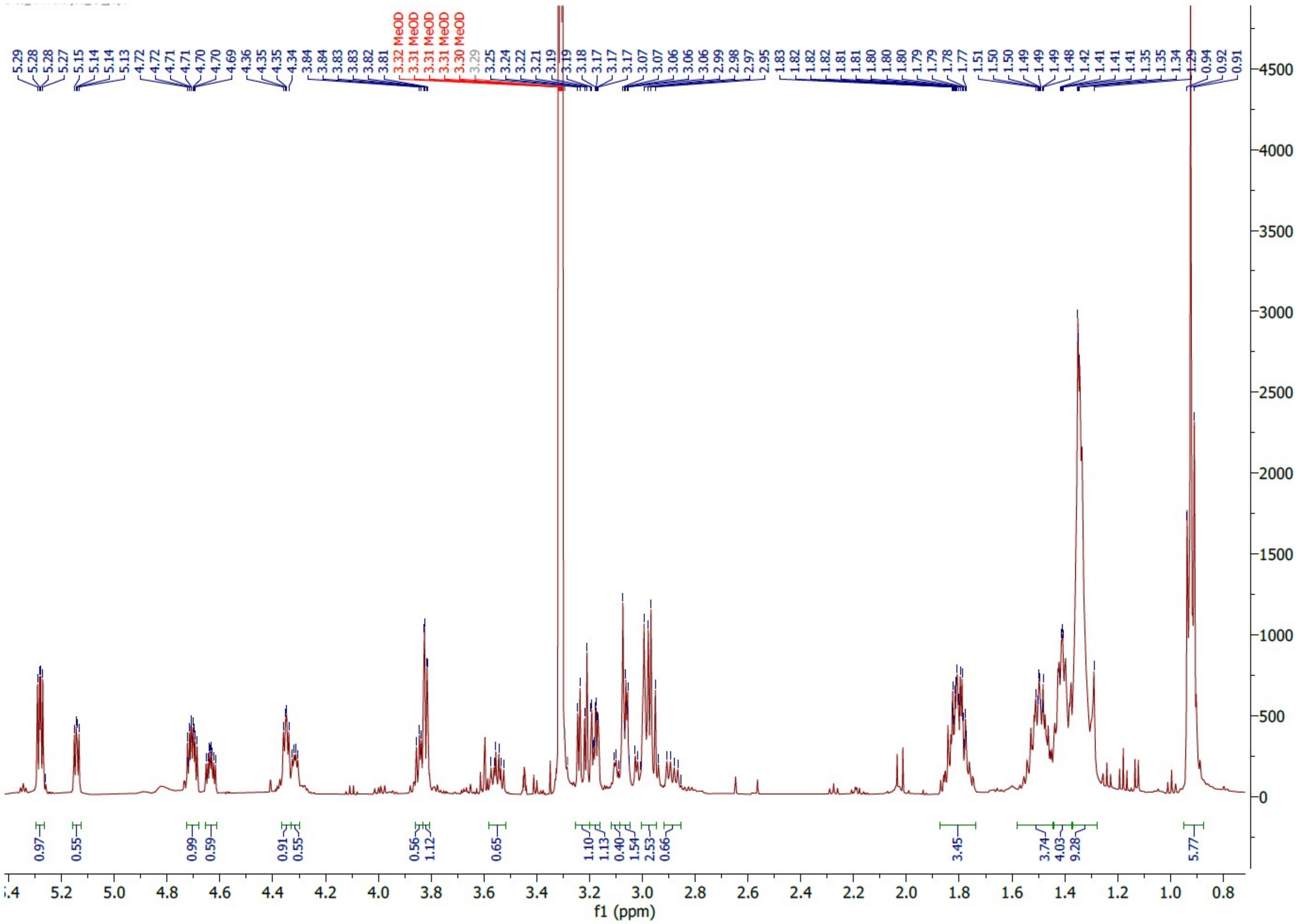

Fig. S 16 ^{1}H NMR spectrum (500 MHz, MeOD) of synthetic **24** and **25**.

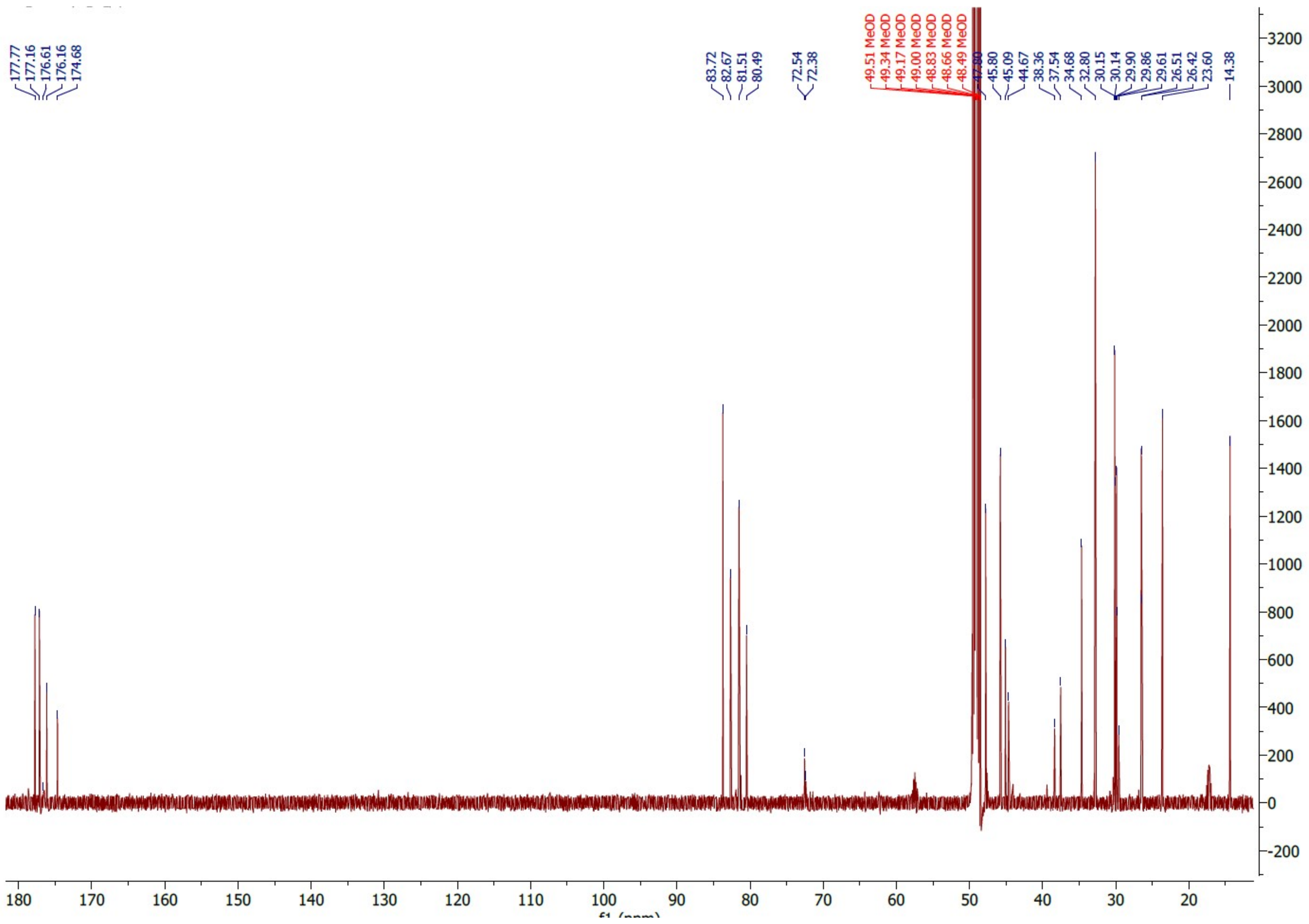

Fig. S 17 ^{13}C NMR spectrum (125 MHz, MeOD) of synthetic **24** and **25**.

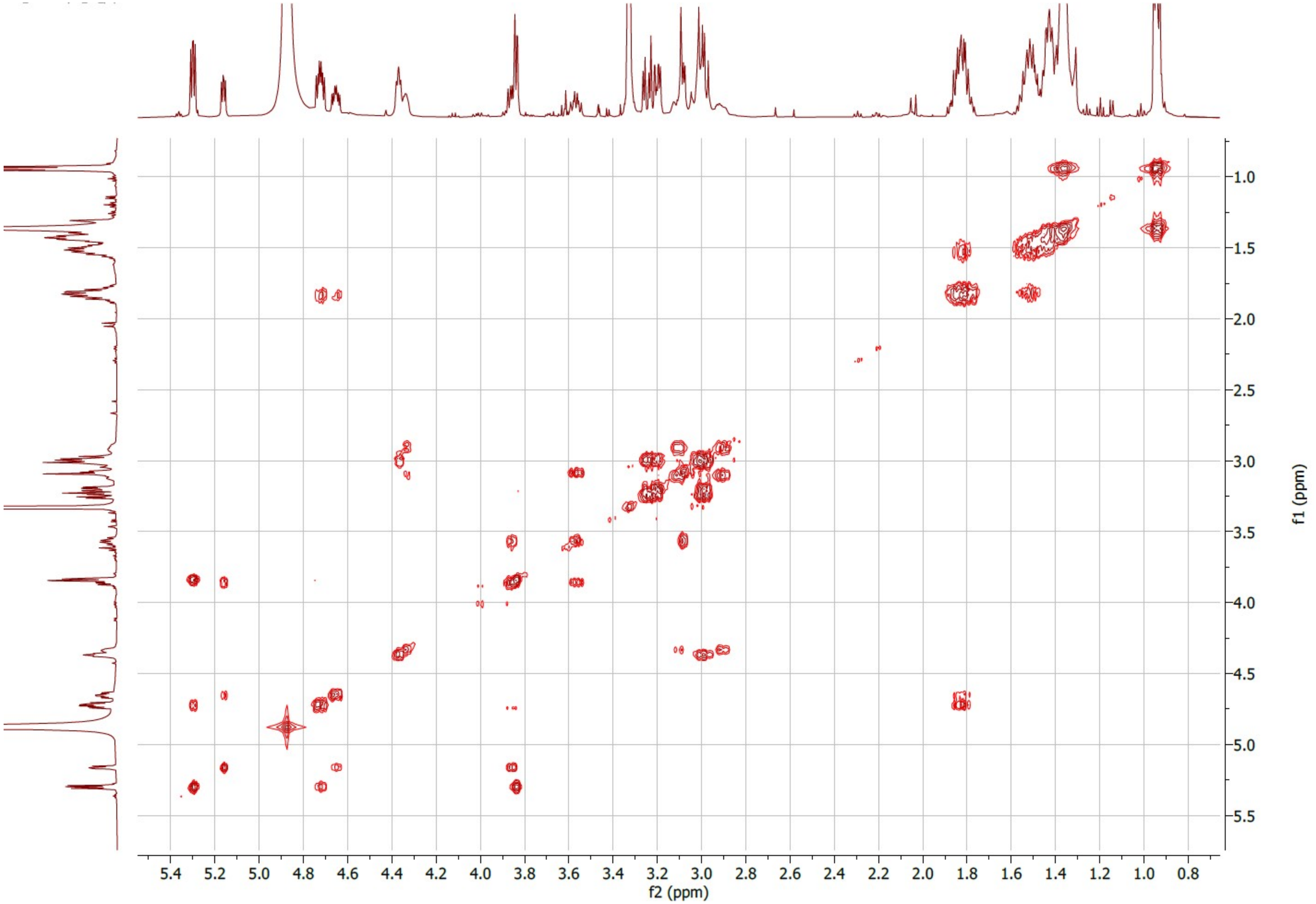

Fig. S 18 $^{1}H/^{1}H$ COSY spectrum (500 MHz, MeOD) of synthetic **24** and **25.**

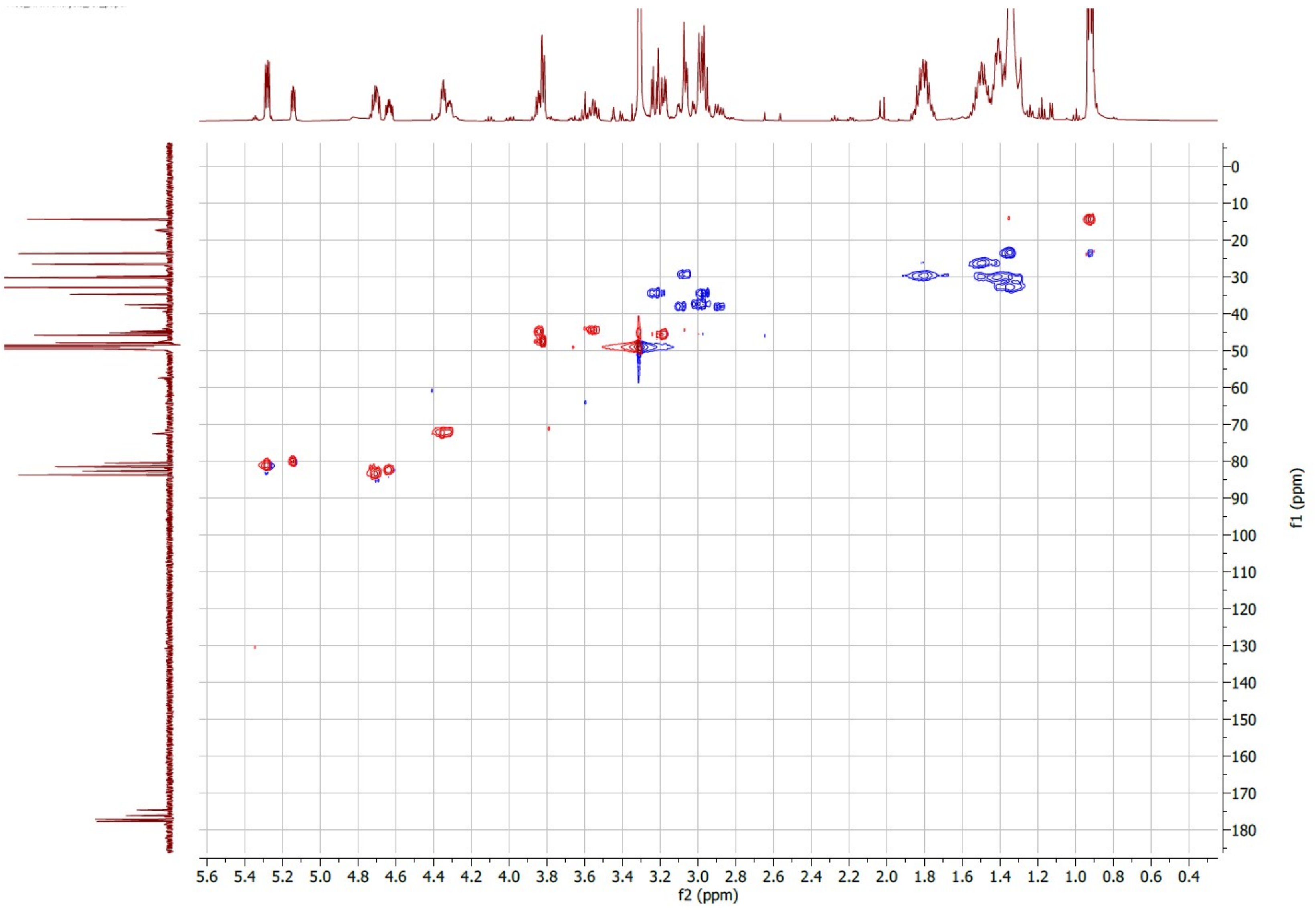

Fig. S 19 $^{1}H/^{13}C$ HSQC spectrum (500 MHz, MeOD) of synthetic **24** and **25.**

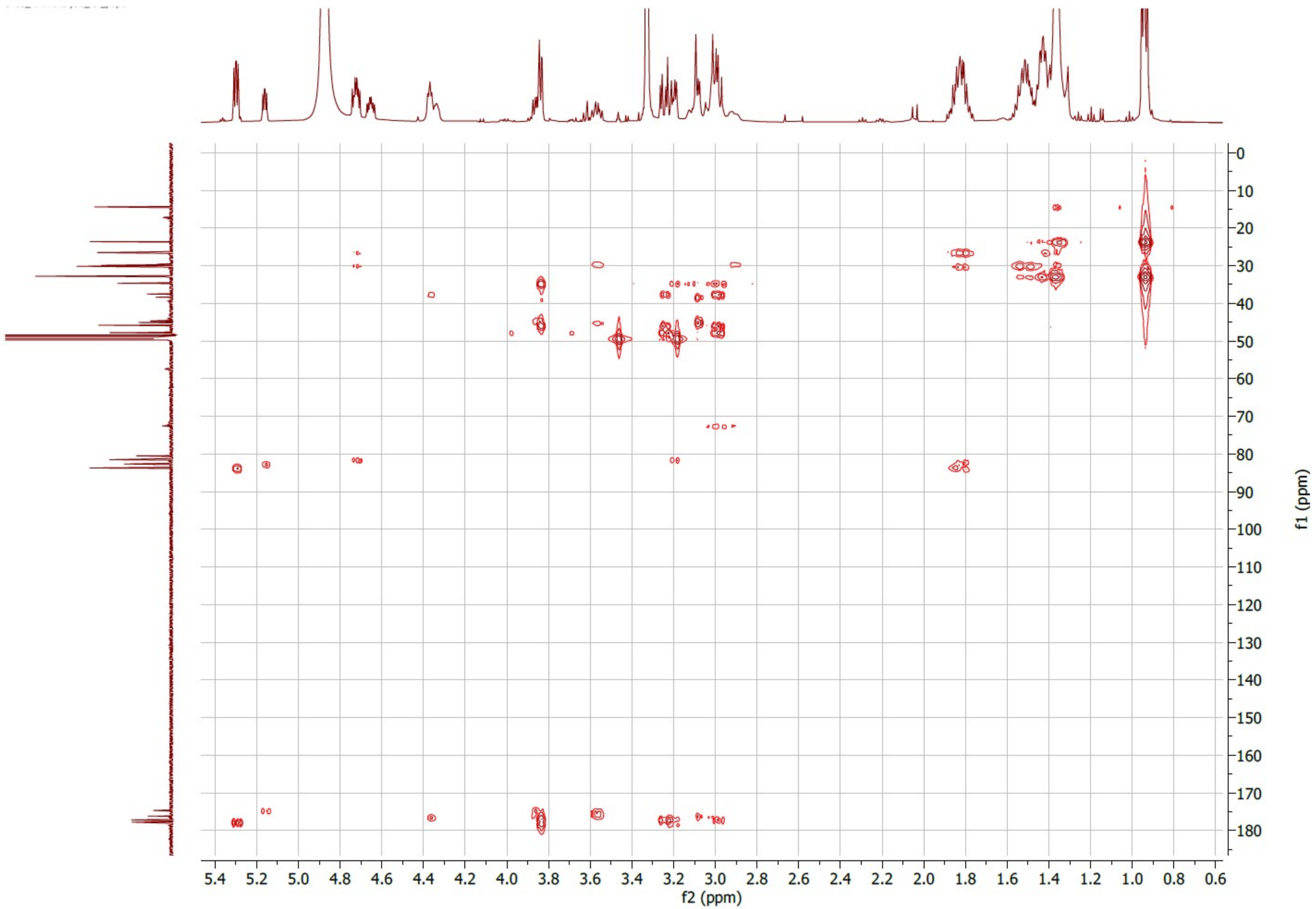

Fig. S 20 $^1H/^{13}C$ HMBC spectrum (500 MHz, MeOD) of synthetic **24** and **25**

Notes and references

1 B. Ferko, M. Zeman, M. Formica, S. Veselý, J. Doháňošová, J. Moncol, P. Olejníková, D. Berkeš, P. Jakubec, D. J. Dixon and O. Caletková, *J. Org. Chem.*, 2019, **84**, 7159–7165.

2 M. G. Schriefer, H. Schrey, H. Zeng, M. Stadler and R. Schobert, *Org. Biomol. Chem.*, 2021, **19**, 4743–4751.

3 F. Surup, E. Kuhnert, E. Lehmann, S. Heitkämper, K. D. Hyde, J. Fournier and M. Stadler, *Mycology*, 2014, **5**, 110–119.

4 K. Becker, A. Wessel and J. J. Luangsa-ard, 1–11.

5 H. Zeng, M. Stadler, W. Abraham, M. Müsken and H. Schrey, .
